# Large-scale analysis reveals that the genome features of simple sequence repeats are generally conserved at the family level in insects

**DOI:** 10.1186/s12864-017-4234-0

**Published:** 2017-11-06

**Authors:** Simin Ding, Shuping Wang, Kang He, Mingxing Jiang, Fei Li

**Affiliations:** 1 Ministry of Agriculture Key Lab of Molecular Biology of Crop Pathogens and Insects, Zhejiang University, 866 Yuhangtang Road, Hangzhou, 310058 China; 2 Technical Centre for Animal Plant and Food Inspection and Quarantine, Shanghai Entry-exit Inspection and Quarantine Bureau, Shanghai, 200135 China

**Keywords:** Insect, SSR, Genome features, Phylogenetic analysis, Taxon, Molecular marker

## Abstract

**Background:**

Simple sequence repeats (SSR), also called microsatellites, have been widely used as genetic markers, and have been extensively studied in some model insects. At present, the genomes of more than 100 insect species are available. However, the features of SSRs in most insect genomes remain largely unknown.

**Results:**

We identified 15.01 million SSRs across 136 insect genomes. The number of identified SSRs was positively associated with genome size in insects, but the frequency and density per megabase of genomes were not. Most insect SSRs (56.2−93.1%) were perfect (no mismatch). Imperfect (at least one mismatch) SSRs (average length 22−73 bp) were longer than perfect SSRs (16−30 bp). The most abundant insect SSRs were the di- and trinucleotide types, which accounted for 27.2% and 22.0% of all SSRs, respectively. On average, 59.1%, 36.8%, and 3.7% of insect SSRs were located in intergenic, intronic, and exonic regions, respectively. The percentages of various types of SSRs were similar among insects from the same family. However, they were dissimilar among insects from different families within orders. We carried out a phylogenetic analysis using the SSR frequencies. Species from the same family were generally clustered together in the evolutionary tree. However, insects from the same order but not in the same family did not cluster together. These results indicated that although SSRs undergo rapid expansions and contractions in different populations of the same species, the general genomic features of insect SSRs remain conserved at the family level.

**Conclusion:**

Millions of insect SSRs were identified and their genome features were analyzed. Most insect SSRs were perfect and were located in intergenic regions. We presented evidence that the variance of insect SSRs accumulated after the differentiation of insect families.

**Electronic supplementary material:**

The online version of this article (10.1186/s12864-017-4234-0) contains supplementary material, which is available to authorized users.

## Background

Simple sequence repeats (SSR), also known as microsatellites, are tandem repetitions of 1–6 bp motifs that are found in all eukaryotic genomes [1]. SSRs are mainly distributed in noncoding regions, but are also found in coding regions [2]. Some studies have indicated that SSRs are preferentially associated with retrotransposons [3]. Due to replication slippage [4] and unequal crossing-over during meiosis [5–7], SSRs have undergone rapid expansions and contractions, leading to variation in SSR length among populations of a single species. Because of these characteristics, SSRs have been widely used as molecular markers for fingerprinting, parentage analysis, genetic mapping, and analysis of genome structure [8–12]. Moreover, numerous studies suggest that SSRs may have biological functions and evolve in a complex process under selective pressure [11, 13, 14]. For example, the expansion of a dinucleotide SSR (AC repeat) in the promoter region of *CYP6CY3*, a P450 gene, resulted in its overexpression, allowing a tobacco-adapted race of polyphagous aphid (*Myzus persicae*) to increase its ability to detoxify nicotine [15].

Insects are one of the most diverse animal classes on our planet. Microsatellite markers are highly polymorphic and selectively neutral [16, 17], and thus are powerful genetic tools to investigate the spatial and temporal population dynamics and evolutionary trends of insects. So far, SSR diversity has been extensively surveyed in more than 200 insects to validate their use as molecular markers to infer the demography and relationships of closely related populations or species [10, 18, 19]. A comparative analysis of SSRs occurring within protein-coding regions of 25 insect species suggested that these repeats represent characteristic features of insect genome diversity [13]. Investigation of imperfect microsatellites (at least one mismatch) in five taxonomic orders (Diptera, Hymenoptera, Lepidoptera, Coleoptera, and Hemiptera) revealed their evolutionary paths across genomes [20]. However, the diversity, features, and evolutionary significance of microsatellites in insects are far from being fully understood. Here, we investigated SSRs in 136 insect species, representing 16 taxonomic orders. Millions of insect SSRs were identified from these insects. Cluster and divergence analysis based on the frequencies of various SSRs were also performed.

## Results

### The number, density and relative abundance of SSRs in 136 insect genomes

**Fig. 1:**
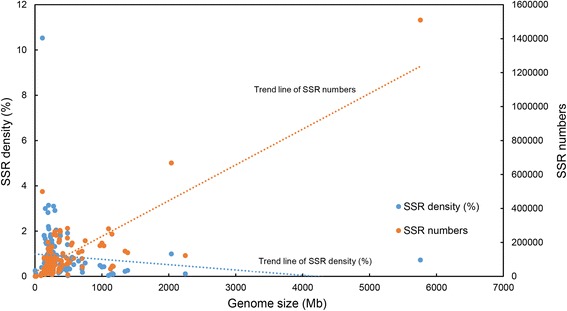
The relationship between SSR number, SSR density, and genome size. The number of identified SSRs was positively associated with genome size

We identified a total of 15.01 million SSRs from 136 insect genomes (Additional file 1: Table S1). Typically, SSRs represent only a very small proportion of insect genomes, ranging from 0.02% to 3.1% of the whole genome (Additional file 1: Table S1, Additional file 2: Fig. S1). Interestingly, we found that the body louse (*Pediculus humanus*) had a much higher percentage (10.53%) of SSRs. Some insects had very few SSRs, such as the cochineal insect *Dactylopius coccus* (225 SSRs), the small green stink bug *Piezodorus guildinii* (432 SSRs), and the beetle *Priacma serrata* (859 SSRs). In contrast, over 500,000 SSRs were identified in some insects, including the body louse *Pediculus* *humanus*, the German cockroach *Blattella germanica*, and the locust *Locusta migratoria* has the largest animal genome so far [21] and was also shown to have the largest number of SSRs (1.5 million in total). The number of SSRs is positively correlated with genome size (Spearman’s rho = 0.499, *P* < 0.001), indicating that the abundance of SSRs varies greatly with insect species (Fig. 1).

SSR density (total bases of SSR (in bp) per Mb of genome) had a significantly negative relationship with genome size (Spearman’s rho = −0.228, *P* = 0.007) (Fig. 1). The density was significantly positively correlated with genome GC content, but the correlation was not strong (Spearman’s rho = 0.183, *P* = 0.033). Interestingly, the SSR frequency in various genomes (i.e., number of SSRs per Mb genome) varied tremendously in insects, ranging from 12 to 4509 (mean 251). The highest frequency (4509) was found in *P. humanus*, followed by the cactophilic fruitfly, *Drosophila mojavensis* (1038). SSR frequency was significantly negatively correlated with genome size (Spearman’s rho = −0.191, *P* = 0.026) and significantly positively correlated with genome GC content (Spearman’s rho = 0.178, *P* = 0.038), but none of the correlations was strong.

Most identified SSRs (56.2−93.1%) were perfect. The length of imperfect microsatellites (range 22−73 bp, average 34.8 bp) was significantly higher (t = −22.834, df = 175.875, *P* < 0.001) than that of perfect SSRs (range 16−30 bp, average 20.4 bp) in each species. To evaluate the relationship between SSR length and motif imperfection, we determined the frequency of mismatches in each locus. Imperfect SSRs contained 0.43−3.23% (1.85% on average) motif mismatches, which mainly appeared in the SSRs with a length of approximately 35 bp (Additional file 3: Table S2). Some closely related species had similar percentages of imperfect SSRs while other closely related species had very different numbers of SSRs. For example, the percentages of imperfect SSRs in 23 *Drosophila* species were very different, ranging from 23% to 44% [13, 20, 22]. In contrast, the imperfect SSR frequencies in two *Nasonia* species were the same at 21%. Similar phenomena were observed in three *Papilio* species (15–16%) and in three *Batrocera* species (22–24%).

### Abundance of SSR motif types

**Fig. 2:**
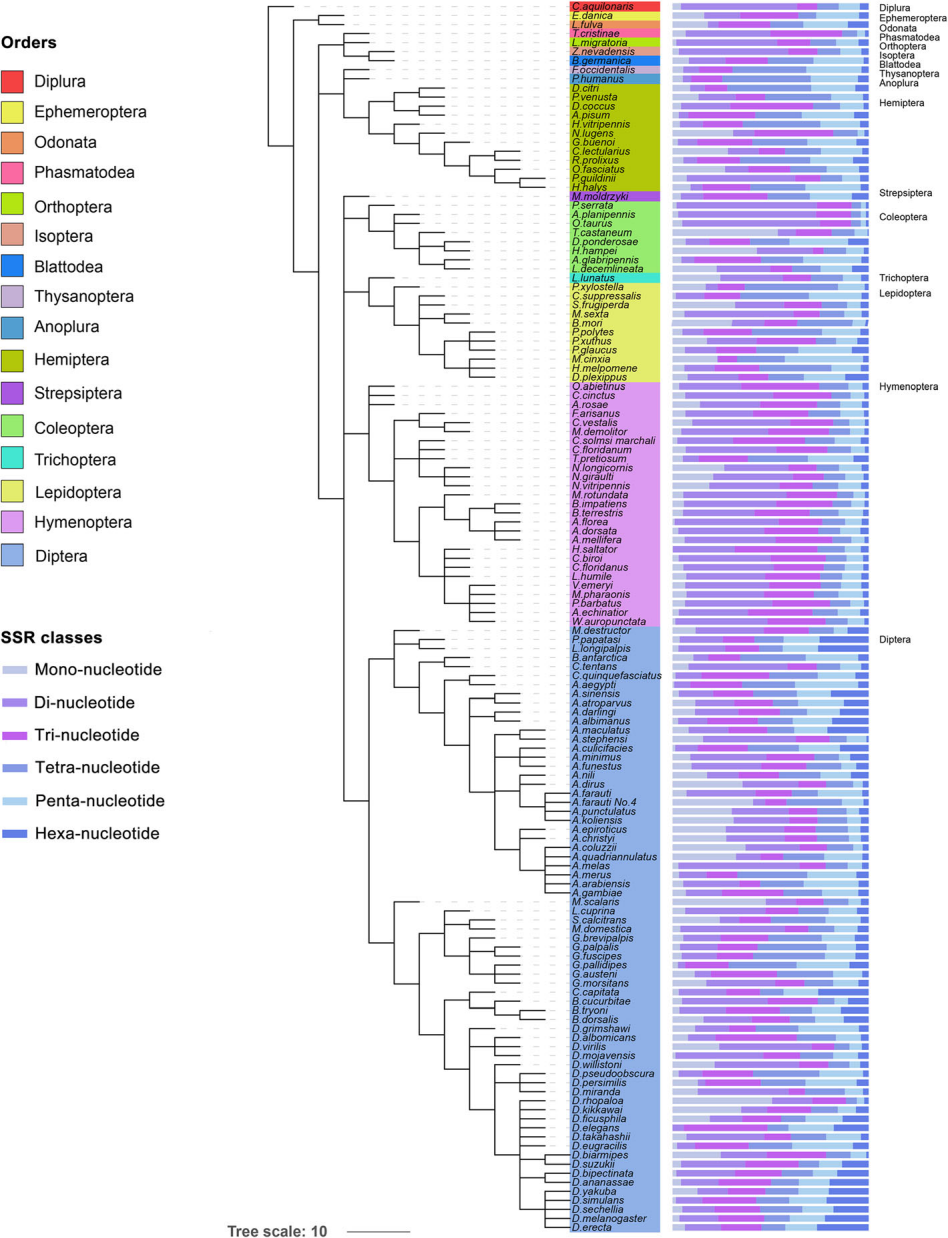
The relative percentages of six SSR motif types within 136 insect genomes. The percentages of mono-, di-, tri-, tetra-, penta- and hexanucleotide repeats are shown in different colors

When comparing the number of various classes of SSRs within genomes, we found that the percentages of di- and trinucleotide SSRs (27.2% and 22.0% on average, respectively) were significantly higher (*P* < 0.05, Tukey test) than those observed for mono- (11.6%), tetra- (17.9%), penta- (14.6%), and hexa-nucleotide repeat types (6.7%) (Additional file 4: Table S3, Fig. 2).

Among mononucleotide repeats, the A/T type was predominant, accounting for 10.3% of the repeat motifs. AG/GA/CT/TC and AC/CA/GT/TG were the most frequent dinucleotide SSRs motifs, accounting for 10.2% and 10.0%, respectively. The next most abundant type was the sequences with AT/TA (6.6%). Among trinucleotide repeats, the AAT/ATA/TAA/ATT/TAT/TTA motif was most abundant (6.3%), and each of the other repeat types accounted for less than 3% (Additional file 5: Table S4).

**Fig. 3:**
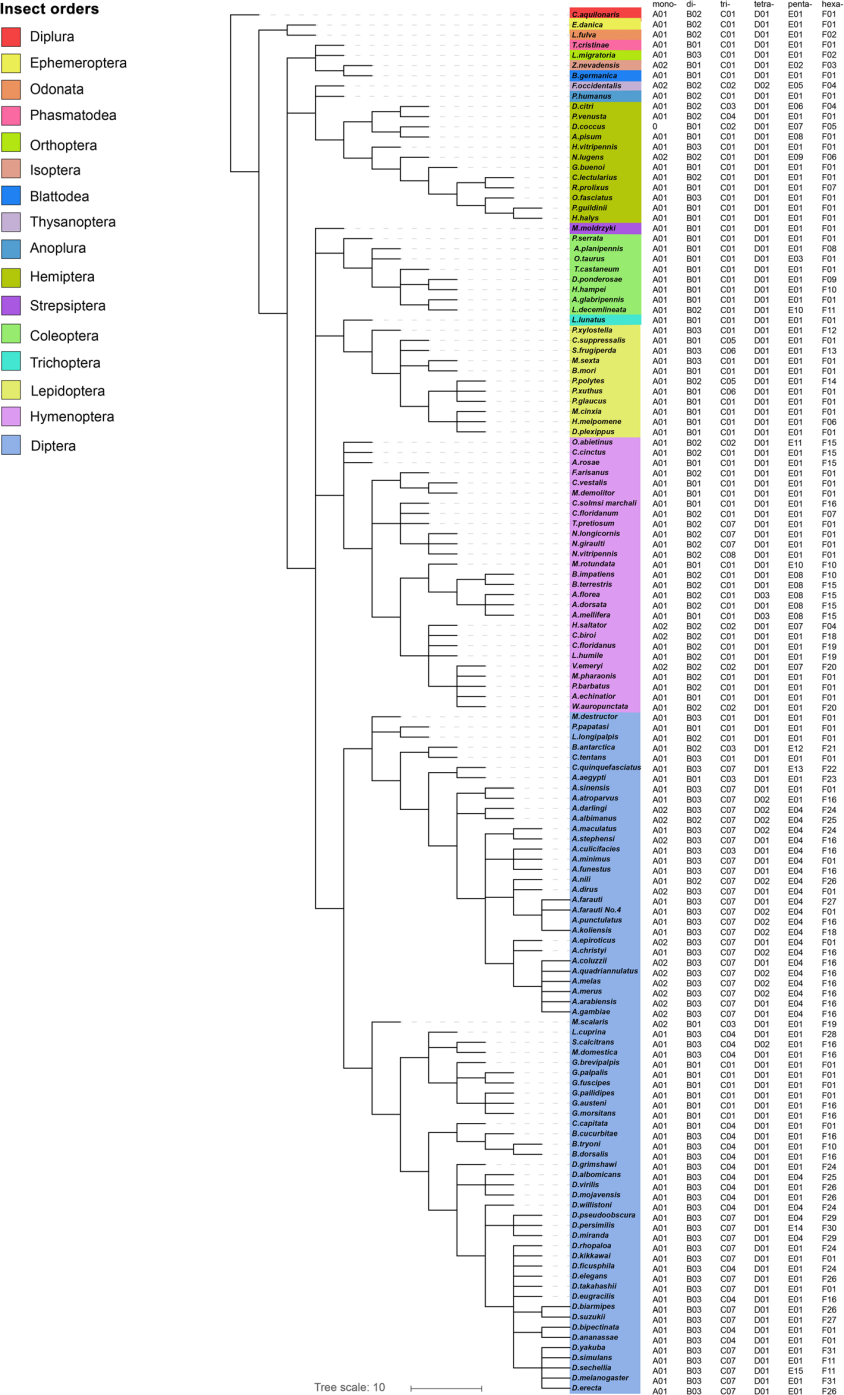
SSR abundance across insect genomes. Capital letters followed with number (A01 etc.) stand for different motif sequences as given in Additional file 3: Table S2. Zero means no SSRs of this class were identified

Between the two types of monomer repeats, the A/T type was significantly more abundant (82.4%) than the G/C type (17.6%) (*t* = 22.962, df = 268, *P* < 0.001). Analysis of all dinucleotide repeats revealed that the GC/CG type accounted for only 0.9%, significantly lower (*P* < 0.05, Tukey test) than each of the other three types (AT/TA, AG/GA/CT/TC and AC/CA/GT/TG, each 30.5−36.4%). Among the trinucleotide SSR repeats, those containing two continuous Gs or Cs accounted for only 3.8−4.3%, which was significantly lower (*P* < 0.05, Tukey test) than other trinucleotide types (6.9−30.0%) (Additional file 6: Table S5). These data indicated that most SSRs in insect genomes consist of AT bases. AT-rich SSR containing motifs, such as AAT/AAAT/AAAAT/AAAAAT or ATA/ATAT/AATAAT/AAAATA, were very common (Fig. 3, Additional file 7: Table S6).

### SSR diversity in different insect taxa

We analyzed the relative abundance of various types of SSRs in different insect taxa, and found that the frequencies of some SSR classes were different at the order level. For example, dinucleotide SSRs (the most abundant of the six types as revealed in Additional file 4: Table S3) accounted for 42.6% on average (range 13.2−71.5%) in the genomes of Hymenoptera, which was significantly higher than that observed in Hemiptera (18.2%), Lepidoptera (12.8%), and Coleoptera (9.2%) (*P* < 0.05, Tukey test) and also higher than that in Diptera (27.5%) (Fig. 2). At the family level, high conservation was observed in terms of the relative abundance of various types of SSRs. This was the most obvious for the families Cupedidae, Buprestidae, and Scarabaeinae that belong to the order Coleoptera. In addition, when viewed at the genus level, SSR frequency was also quite similar among genera within families (Fig. 2). The conservation of SSRs at the family level was also confirmed by analysis of the most abundant motifs. The results indicated that the most abundant motifs were conserved at the family level (Fig. 3).

At the species level, relative abundance of SSRs was very similar within some genera, such as *Apis* of Apidae, *Nasonia* of Pteromalidae, *Anopheles* of Culicidae, and *Glossina* of Glossinidae. However, interspecies differences in SSR frequency were also observed in some genera, such as *Papilio*, *Drosophila*, and *Anopheles* (Fig. 2). Taken together, the evolution of SSR diversity varied depending on the insect species, suggesting that insect SSRs face dissimilar selection pressures in different taxa.

### Distribution of SSRs in different genomic regions

On average, 59.1% (range 49.6−62.0%) of SSRs were distributed in intergenic regions. Within genes, SSRs were mainly found in introns, which accounted for 36.8% (range 27.8−49.9%) of total SSRs, while only 3.7% (range 0.3−9.9%) were in exons (Table 1, Additional file 8: Table S7 and Additional file 9: Table S8). We compared the occurrence of SSRs in different genomic regions between Diptera and Hymenoptera genomes. In Diptera, the exonic SSRs reached 5.5% (range 1.2−9.95%), which was significantly higher than the 2.4% (range 0.3−5.8%) observed in Hymenoptera (*t* = 5.608, *df* = 36.312, *P* < 0.001). By contrast, the percentage of intronic SSRs were significantly lower in Diptera (average 33.2%) when compared to Hymenoptera (average 45.2%) (*t* = −2.296, *df* = 32.546, *P* = 0.028). The SSRs that occurred in mRNA regions were not significantly different (*t* = −1.758, *df* = 30.034, *P* = 0.089), accounting for 38.9% and 47.8% in Diptera and Hymenoptera, respectively.

**Table 1:** Total number of SSRs in different genome regions

Order	Species	Exon	Intron	Intergenic regions	Spanning exon-intron	Spanning intergenic-genetic	total number
Anoplura	*P. humanus*	3797	82,101	377,312	340	287	463,837
Coleoptera	*D. ponderosae*	275	1726	6182	23	10	8216
	*T. castaneum*	734	7623	9493	43	21	17,914
Diptera	*A. aegypti*	4510	272,342	91,215	537	50	368,654
	*A. coluzzii*	3896	22,894	52,062	127	95	79,074
	*A. darlingi*	27,936	45,779	213,703	2749	586	290,753
	*A. gambiae*	4781	28,746	74,093	108	106	107,834
	*A. sinensis*	3219	5862	23,306	75	30	32,492
	*A. stephensi*	4125	11,654	54,969	112	488	71,348
	*B. cucurbitae*	2388	42,930	40,223	46	161	85,748
	*B. dorsalis*	1903	27,991	29,792	37	77	59,800
	*C. capitata*	3862	113,867	100,518	73	288	218,608
	*C. quinquefasciatus*	10,339	53,060	221,906	1283	396	286,984
	*D. ananassae*	3823	15,074	32,900	73	26	51,896
	*D. erecta*	3810	10,660	23,757	49	27	38,303
	*D. grimshawi*	8444	47,707	95,949	172	70	152,342
	*D. melanogaster*	2201	30,075	15,571	56	95	47,998
	*D. mojavensis*	8222	56,598	124,059	203	69	189,151
	*D. persimilis*	5613	34,289	62,326	172	76	102,476
	*D. pseudoobscura*	6227	32,734	67,334	137	65	106,497
	*D. sechellia*	2527	9763	20,328	63	36	32,717
	*D. simulans*	2253	9234	19,388	57	24	30,956
	*D. virilis*	8114	39,513	88,738	140	60	136,565
	*D. willistoni*	6731	45,312	97,124	142	59	149,368
	*D. yakuba*	3571	13,101	28,165	79	35	44,951
	*M. destructor*	3883	17,407	59,110	519	415	81,334
	*M. scalaris*	268	770	2581	25	7	3651
	*M. domestica*	2298	58,145	64,685	45	227	125,400
Hemiptera	*A. pisum*	2349	93,793	71,989	164	511	168,806
	*D. citri*	459	78,562	78,172	115	179	157,487
	*N. lugens*	955	9002	25,103	73	35	35,168
	*R. prolixus*	505	24,038	87,771	42	45	112,401
Hymenoptera	*A. dorsata*	1083	83,173	59,480	119	334	144,189
	*A. florea*	1484	81,374	60,290	123	368	143,639
	*A. mellifera*	4455	82,325	133,229	724	397	221,130
	*A. rosae*	973	54,968	41,550	60	240	97,791
	*B. impatiens*	890	27,439	14,460	43	260	43,092
	*B. terrestris*	892	25,178	13,437	27	267	39,801
	*C. floridanus*	3493	22,024	92,188	682	227	118,614
	*C. biroi*	1446	30,066	14,794	57	179	46,542
	*C. solmsi marchali*	1579	115,235	126,743	424	985	244,966
	*F. arisanus*	744	8932	4937	32	54	14,699
	*H. saltator*	12,331	79,960	388,788	2777	818	484,674
	*L. humile*	1635	6724	41,722	67	61	50,209
	*M. rotundata*	1018	13,356	7637	35	84	22,130
	*M. demolitor*	2343	60,307	44,657	186	553	108,046
	*N. vitripennis*	1897	39,230	70,786	175	24	112,112
	*P. barbatus*	1860	13,131	78,291	119	97	93,498
	*V. emeryi*	1787	33,971	22,043	59	304	58,164
	*W. auropunctata*	1724	51,892	36,798	64	345	90,823
	*A. echinatior*	4443	35,426	115,736	790	203	156,598
Isoptera	*Z. nevadensis*	879	16,054	48,801	61	27	65,822
Lepidoptera	*B. mori*	501	12,750	76,795	41	21	90,108
	*C. suppressalis*	861	5346	8584	157	6	14,954
	*D. plexippus*	553	11,184	24,726	40	18	36,521
	*H. melpomene*	571	13,315	23,606	45	45	37,582
	*M. sexta*	864	17,324	24,138	40	67	42,433
	*P. xylostella*	9700	74,901	144,119	1862	291	230,873

### Evolution analysis of insect SSR

**Fig. 4:**
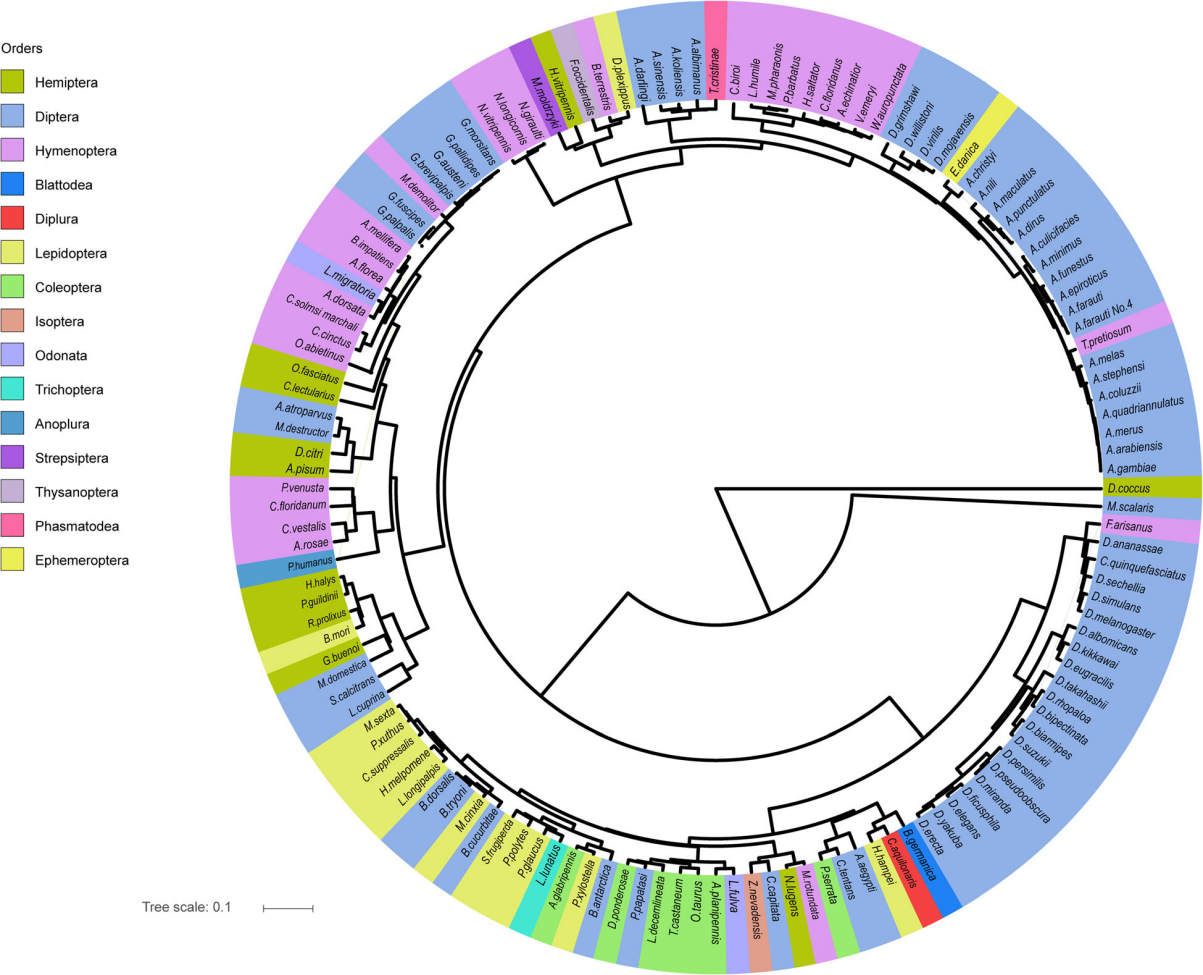
Phylogenetic analysis of insect SSRs. Symmetrized Kullback-Leibler divergence analysis was used and the evolutionary tree was constructed using the UPGMA method with MEGA6 software, and viewed with ITOL software

Clustering analysis showed that the frequencies of various SSRs were largely similar within different insect orders (Fig. 4). A symmetrized Kullback-Leibler divergence analysis, based on the percentage of dinucleotide combinations, could almost perfectly separate Hymenoptera and Diptera from other insects (Additional file 10: Fig. S2). For Diptera, 54 species (80.6% of the total) clustered into two branches: 1) Diptera-I, which contained only flies of most families; and 2) Diptera-II, which was comprised solely of mosquitoes. Most Dipteran families can be readily separated from others, except for several Drosophilidae species (Additional file 11: Fig. S3). All hymenopteran species, except for *Cotesia vestalis* and *Microplitisde molitor,* clustered together (Additional file 10: Fig. S2). Similar results were obtained when the analysis was carried out using the tri-, tetra-, and penta-nucleotide motif information (Additional file 12: Fig. S4, Additional file 13: Fig. S5, Additional file 14: Fig. S6). In general, most insects were clearly divided using SSR frequencies at the family level, but not at the order level.

## Discussion

Here, we identified millions of SSRs in 136 insect genomes and analyzed their features. The abundance and densities of insect SSRs were correlated with genome sizes. However, no strong correlation was found between the SSR density and GC content, nor was there a strong correlation between SSR frequency and genome size. This pattern was also observed in Tombusviridae [23], potexvirus [24] and carlaviruses [25]. In contrast, SSR frequency was shown to be inversely related to genome sizes in plants [26], Bovid species [27], fungi [28], and maize [29]. Both SSR frequency and density were negatively correlated with GC-content in these species.

SSRs only account for a very small proportion of insect genomes, and they generally do not have clear functions. Therefore, SSRs are believed to face relatively low selection pressures and accumulate mutations faster than coding genes [11]. SSRs have been widely used as genetic markers to distinguish individual insects from geographically distinct populations [30, 31]. Phylogenetic analysis of 136 insect SSRs indicated that the evolutionary tree constructed with SSR genome features was largely inconsistent with species trees, especially at the family level. This suggested that too many mutations have accumulated in insect SSRs since the division of the insect families. However, we found that insect species belonging to the same family tended to cluster together in the evolutionary tree, suggesting that selection pressures of SSRs were maintained at the family level. Thus, insect SSRs are good molecular markers to distinguish closely related insect species.

We found that perfect SSRs were significantly more abundant than imperfect SSRs in insects. Overall, perfect SSRs accounted for 56−93% of the identified microsatellites, whereas imperfect SSRs made up only 7−44%. This is consistent with similar results in plants, such as the Triticeae species [32], and previous reports in insects [13, 20]. The frequency of forming mismatch motifs varied with the length of the motif. For mono-, penta-, and hexanucleiotide SSRs, <19% were imperfect. In contrast, in di- and trinucleotide SSRs, ~31% of motifs were imperfect. This pattern was conserved in almost all tested insects, suggesting that this is a conserved feature in insect microsatellites [20]. A large fraction of the trinucleotide SSRs were derived from codon repeats, and the occurrence of these trinucleotide mismatches contributed to codon bias in the insect genomes [13, 20].

In insects, nearly three fifths of SSRs were found in intergenic regions, consistent with previous reports. Only 3.7% (0.3−9.9%) of SSRs occurred in exonic regions, which can be attributed to negative selection against frameshift mutations in coding regions [33]. In contrast, intronic SSRs accounted for 36.8%, which is 10-fold higher than exonic SSRs. It has been reported that intronic SSRs may affect gene expression [34], suggesting that the functions of intronic SSR will require more exploration.

## Conclusions

In this study, we carried out a comprehensive analysis of SSRs in 136 insects. This is the first large-scale analysis of insect SSRs, and included more than 100 insect species. The results confirmed some previous conclusions about insect SSRs. The numbers of insect SSRs were positively associated with the genome sizes whereas the frequency and density were not. Both phylogenetic analysis and most abundant motif analysis showed that the insect SSRs were generally evolutionary conserved at the family level but not at the order level.

## Methods

### Genome sequences

At present, the genomes of 136 insect species are publically available, including 67 Diptera species, 27 Hymenoptera species, 12 Hemiptera species, 11 Lepidoptera species, eight Coleoptera species, and one species each of Diplura, Ephemeroptera, Odonata, Strepsiptera, Trichoptera, Thysanoptera, Anoplura, Phasmatodea, Orthoptera, Isoptera, and Blattodea (Additional file 1: Table S1). The genome sequences of all insects were downloaded from InsectBase 1.0 [35]. Ambiguous nucleotides were removed from the genomes prior to analysis.

### Identification of insect SSRs

SSRs were identified using the SciRoKo 3.4 using default parameters [36]. According to the motifs, the repeat sequences were divided into six classes: mono-, di-, tri-, tetra-, penta-, and hexa-nucleotide SSRs [20]. For each class, only sequences with a length of ≥15 nucleotides were considered as SSRs. Briefly, SSRs with no mismatch in the motif were defined as perfect SSRs, while SSRs with at least one mismatch in the motif were defined as imperfect SSRs. The criteria used for defining imperfect SSRs was as following: ≥ 30 bp SSRs with 1–3 mismatches and ≤30 bp SSRs with ≥3 mismatches.

### Analysis of SSRs

We calculated the frequency and density of SSRs in each of the available insect genomes. The frequency was determined as the percentage of the total number of SSRs per megabase (Mb) of genome sequence. The relative density was determined as the length (in bp) of SSRs sequences in the total Mb of genomic sequence analyzed. The relative abundances of perfect and imperfect repeat classes were calculated within each class of SSR, and their size distribution range and mean lengths were calculated. Associations between SSR number, frequency, and density with the genome sizes and GC contents were tested using Spearman rank correlation to determine whether there was significant correlation between the two variables (IBM SPSS Statistics, 2011).

### Microsatellite distribution in insect genomes

Among the 136 insect species, 58 genomes were annotated with protein-coding genes accompanied by gff3 annotation files containing the positional information on exons and introns. The distribution of SSRs in these different regions was determined by mapping the SSRs to the genome using a Perl script.

### Evolutionary analysis of insect SSRs

We constructed phylogenetic trees using insect SSRs with symmetrized Kullback-Leibler divergence analysis [37, 38]. The differences between two species were measured quantitatively with the percentages of SSRs ([p(x) and q(x)] in two species respectively, where x represents the class of SSR (di-, tri-, tetra-, penta-, and hexa-nucleotide repeat types). All pairwise comparisons among the 136 insect species were performed. Cluster analysis was performed using the UPGMA method [39] with the MEGA6 software package. Phylogenetic trees were visualized with ITOL software (http://itol.embl.de/) [40].

## Additional files

Additional file 1: **Table S1.** The genome sizes, GC content, SSR numbers and densities of 136 insect genomes. (DOCX 40 kb)

Additional file 2: **Figure S1.** The insect genome sizes and SSR densities of 136 insects, showing that SSR densities have no relationship with genome size. (TIFF 2488 kb)

Additional file 3: **Table S2.** Perfect and imperfect SSRs in insect genomes. (DOCX 36 kb)

Additional file 4: **Table S3.** Percentage (%) of different types of SSRs. (DOCX 14 kb)

Additional file 5: **Table S4.** Percentage of different types of SSRs in all SSRs (DOCX 15 kb)

Additional file 6: **Table S5.** Percentage of different types of SSRs calculated within classes. (DOCX 15 kb)

Additional file 7: **Table S6.** The sequences of SSR motifs. (DOCX 21 kb)

Additional file 8: **Table S7.** Relative abundance of perfect SSRs in different genomic regions. (DOCX 24 kb)

Additional file 9: **Table S8.** Relative abundance of imperfect SSRs in different genomic regions. (DOCX 26 kb)

Additional file 10: **Figure S2.** Phylogenetic analysis of 136 insect genomes using the relative abundance information from di-nucleotide SSRs. (TIFF 2484 kb)

Additional file 11: **Figure S3.** Phylogenetic analysis of Diptera using the relative abundance information from six types of SSRs, showing that dipteran insects can be clearly classified at the family level. (TIFF 1521 kb)

Additional file 12: **Figure S4.** Phylogenetic analysis of 136 insect genomes using the relative abundance information from tri-nucleotide SSRs (TIFF 1694 kb)

Additional file 13 **Figure S5.** Phylogenetic analysis of 136 insects using the relative abundance information from tetra-nucleotide SSR. (TIFF 2340 kb)

Additional file 14: **Figure S6.** Phylogenetic analysis of 136 insects using the relative abundance information from penta-nucleotide SSR. (TIFF 2093 kb)

## Abbreviations

Bp: base pair
di-: dinucleotide
gff3: generic Feature Format3
hexa-: hexanucleotide
mb: megabase
mono-: mononucleotide
penta-: pentanucleotide
SSR: simple sequence repeats
tetra-: tetranucleotide
tri-: trinucleotide
