# Supplementary material for: Large-scale analysis reveals that the genome features of simple sequence repeats are generally conserved at the family level in insects
Source: BMC Genomics. 2017 Nov 6;18:848. doi: 10.1186/s12864-017-4234-0 (PMC5674736; doi:10.1186/s12864-017-4234-0)
Supplement: Supplementary file 1 — Table S1. The genome sizes, GC content, SSR numbers and densities of 136 insect genomes. (DOCX 40 kb) [file 12864_2017_4234_MOESM1_ESM.docx]

**Table S1. The genome sizes, GC content, SSR numbers and densities of 136 insects**

| **Order** | **Species** | **Genome size**  **(Mb)** | **GC content**  **(%)** | **SSR number** | **SSR density (%)** |
| --- | --- | --- | --- | --- | --- |
| Diplura | *C. aquilonaris* | 3021 | 42.95 | 67731 | 0.65 |
| Ephemeroptera | *E. danica* | 4759 | 33.62 | 71403 | 0.41 |
| Odonata | *L. fulva* | 11581 | 36.45 | 61345 | 0.13 |
| Strepsiptera | *M. moldrzyki* | 1557 | 28.69 | 21746 | 0.34 |
| Hymenoptera | *O. abietinus* | 2012 | 45.03 | 20599 | 3.14 |
|  | *C. cinctus* | 1622 | 40.33 | 47048 | 0.85 |
|  | *A. rosae* | 1638 | 41.20 | 103141 | 1.48 |
|  | *C. vestalis* | 1861 | 30.58 | 95842 | 1.43 |
|  | *M. demolitor* | 2505 | 30.43 | 129995 | 1.58 |
|  | *F. arisanus* | 1536 | 38.60 | 16172 | 0.24 |
|  | *H. saltator* | 2968 | 45.16 | 264338 | 2.91 |
|  | *C. biroi* | 2128 | 41.68 | 49183 | 0.56 |
|  | *C. floridanus* | 2349 | 34.24 | 77286 | 0.91 |
|  | *L. humile* | 2198 | 37.66 | 52715 | 0.14 |
|  | *V. emeryi* | 2879 | 42.17 | 66606 | 0.62 |
|  | *M. pharaonis* | 2623 | 37.03 | 101320 | 1.10 |
|  | *P. barbatus* | 2354 | 36.50 | 102605 | 1.42 |
|  | *A. echinatior* | 2975 | 33.64 | 94483 | 0.91 |
|  | *W. auropunctata* | 3241 | 38.66 | 110000 | 0.94 |
|  | *M. rotundata* | 2727 | 36.56 | 24709 | 0.28 |
|  | *B. impatiens* | 2492 | 37.75 | 46380 | 0.68 |
|  | *B. terrestris* | 2487 | 37.51 | 41867 | 0.44 |
|  | *A. florea* | 2305 | 33.77 | 167850 | 1.79 |
|  | *A. dorsata* | 2303 | 31.92 | 162090 | 1.74 |
|  | *A. mellifera* | 2341 | 32.70 | 173351 | 1.85 |
|  | *C. solmsi marchali* | 2771 | 30.36 | 246266 | 3.10 |
|  | *C. floridanum* | 5550 | 36.25 | 196461 | 0.84 |
|  | *T. pretiosum* | 1962 | 39.88 | 107126 | 1.22 |
|  | *N. longicornis* | 2857 | 42.73 | 71773 | 0.64 |
|  | *N. giraulti* | 2836 | 42.87 | 72713 | 0.68 |
|  | *N. vitripennis* | 2951 | 41.71 | 119085 | 1.32 |
| Diptera | *M. scalaris* | 4901 | 33.80 | 9845 | 0.03 |
|  | *G. brevipal.* | 3154 | 31.21 | 242234 | 2.05 |
|  | *G. palpalis* | 3801 | 33.61 | 263589 | 1.79 |
|  | *G. fuscipes* | 3748 | 33.60 | 270623 | 1.87 |
|  | *G. pallidipes* | 3573 | 34.11 | 202587 | 1.51 |
|  | *G. austeni* | 3703 | 34.09 | 269142 | 2.01 |
|  | *G. morsitans* | 3662 | 34.12 | 218832 | 1.57 |
|  | *L. cuprina* | 4705 | 29.74 | 107289 | 0.26 |
|  | *S, calcitrans* | 9712 | 38.85 | 180694 | 0.49 |
|  | *M. domestica* | 7504 | 35.11 | 209899 | 0.59 |
|  | *D. albomicans* | 2536 | 38.40 | 174499 | 1.45 |
|  | *D. virilis* | 2060 | 39.98 | 144815 | 2.10 |
|  | *D. mojavensis* | 1938 | 39.48 | 201309 | 2.82 |
|  | *D. grimshawi* | 2005 | 37.98 | 165417 | 2.21 |
|  | *D. willistoni* | 2355 | 37.25 | 158258 | 1.55 |
|  | *D. pseudoobscura* | 1527 | 45.20 | 111142 | 1.66 |
|  | *D. persimilis* | 1884 | 44.94 | 116774 | 1.44 |
|  | *D. miranda* | 1367 | 44.89 | 105212 | 1.79 |
|  | *D. rhopaloa* | 1974 | 40.07 | 39898 | 0.47 |
|  | *D. kikkawai* | 1643 | 41.37 | 61396 | 0.82 |
|  | *D. ficusphila* | 1524 | 41.92 | 46724 | 0.69 |
|  | *D. elegans* | 1713 | 40.30 | 51107 | 0.70 |
|  | *D. takahashii* | 1821 | 40.00 | 40916 | 0.54 |
|  | *D. eugracilis* | 1569 | 40.89 | 53879 | 0.87 |
|  | *D. biarmipes* | 1694 | 41.81 | 29652 | 0.41 |
|  | *D. suzukii* | 2329 | 40.70 | 38585 | 0.42 |
|  | *D. yakuba* | 1657 | 42.28 | 48856 | 0.69 |
|  | *D. simulans* | 1378 | 42.48 | 36400 | 0.58 |
|  | *D. sechellia* | 1666 | 42.09 | 40407 | 0.57 |
|  | *D. melanogaster* | 1437 | 42.01 | 48689 | 0.81 |
|  | *D. erecta* | 1527 | 42.27 | 39491 | 0.65 |
|  | *D. bipectinata* | 1673 | 41.61 | 42452 | 0.55 |
|  | *D. ananassae* | 2310 | 41.97 | 57935 | 0.59 |
|  | *B. cucurbitae* | 3748 | 35.15 | 93376 | 0.53 |
|  | *B. tryoni* | 5190 | 36.06 | 81005 | 0.33 |
|  | *B. dorsalis* | 4150 | 36.04 | 66722 | 0.32 |
|  | *C. capitata* | 4848 | 35.31 | 225688 | 1.40 |
|  | *M. destructor* | 1852 | 33.17 | 78490 | 0.97 |
|  | *P. papatasi* | 3638 | 33.60 | 34176 | 0.19 |
|  | *L. longipal.* | 1542 | 35.01 | 16213 | 3.00 |
|  | *A. sinensis* | 2208 | 43.85 | 36164 | 0.34 |
|  | *A. atroparvus* | 2243 | 46.35 | 46164 | 0.47 |
|  | *A. darlingi* | 1370 | 48.30 | 110315 | 1.81 |
|  | *A. albimanus* | 1705 | 49.21 | 105365 | 1.45 |
|  | *A. culicifacies* | 2030 | 42.68 | 36885 | 0.36 |
|  | *A. minimus* | 2018 | 42.70 | 31728 | 0.33 |
|  | *A. funestus* | 2252 | 41.59 | 40505 | 0.39 |
|  | *A. maculatus* | 1419 | 44.21 | 39392 | 0.59 |
|  | *A. stephensi* | 2254 | 45.02 | 75854 | 0.73 |
|  | *A. nili* | 983 | 45.72 | 17972 | 0.39 |
|  | *A. farauti* | 1831 | 44.69 | 57735 | 0.75 |
|  | *A. farauti No.4* | 1464 | 44.54 | 44468 | 0.69 |
|  | *A. punctulatus* | 1462 | 45.32 | 47289 | 0.73 |
|  | *A. koliensis* | 1511 | 45.11 | 43566 | 0.60 |
|  | *A. dirus* | 2163 | 46.18 | 73260 | 0.82 |
|  | *A. epiroticus* | 2235 | 43.95 | 58274 | 0.60 |
|  | *A. christyi* | 1727 | 42.74 | 59530 | 0.78 |
|  | *A. coluzzii* | 2245 | 44.38 | 86331 | 0.94 |
|  | *A. quadriannulatus* | 2838 | 44.76 | 99218 | 0.85 |
|  | *A. melas* | 2242 | 44.84 | 109339 | 1.20 |
|  | *A. merus* | 2880 | 44.64 | 105172 | 0.94 |
|  | *A. arabiensis* | 2466 | 44.68 | 97085 | 0.96 |
|  | *A. gambiae* | 2731 | 44.27 | 107817 | 1.06 |
|  | *C.quinquefasciatus* | 5790 | 37.42 | 106746 |  |
|  | *A. aegypti* | 13840 | 38.27 | 139784 | 0.26 |
|  | *B. antarctica* | 896 | 38.88 | 3862 | 0.08 |
|  | *C. tentans* | 2135 | 31.15 | 23624 | 0.21 |
| Trichoptera | *L. lunatus* | 13459 | 34.74 | 148475 | 0.22 |
| Lepidoptera | *P. xylostella* | 3941 | 38.34 | 90559 | 0.50 |
|  | *C. suppressalis* | 3724 | 35.74 | 54652 | 0.28 |
|  | *P. polytes* | 2270 | 33.96 | 30064 | 0.27 |
|  | *P. xuthus* | 2439 | 33.81 | 31756 | 0.29 |
|  | *P. glaucus* | 3748 | 35.37 | 41047 | 0.21 |
|  | *M. cinxia* | 3899 | 32.57 | 93353 | 0.60 |
|  | *H. melpomene* | 2738 | 32.84 | 48726 | 0.39 |
|  | *D. plexippus* | 2486 | 31.60 | 40544 | 0.41 |
|  | *S. frugiperda* | 3580 | 35.73 | 43853 | 0.25 |
|  | *M. sexta* | 3844 | 35.26 | 45406 | 0.25 |
|  | *B. mori* | 4808 | 37.68 | 105270 | 0.48 |
| Coleoptera | *P. serrata* | 121 | 35.94 | 859 | 0.13 |
|  | *A. planipennis* | 3535 | 34.64 | 27676 | 0.15 |
|  | *O. taurus* | 2705 | 33.12 | 21207 | 0.15 |
|  | *D. ponderosae* | 2528 | 35.91 | 10355 | 0.08 |
|  | *H. hampei* | 1513 | 32.28 | 11901 | 0.14 |
|  | *A. glabripennis* | 7077 | 32.74 | 54480 | 0.18 |
|  | *L. decemlineata* | 11702 | 35.63 | 59100 | 0.10 |
|  | *T. castaneum* | 2287 | 33.86 | 17900 | 0.24 |
| Thysanoptera | *F. occidentalis* | 4158 | 50.87 | 134696 | 0.91 |
| Anoplura | *P. humanus* | 1108 | 27.47 | 499582 | 10.53 |
| Hemiptera | *H. vitripennis* | 22464 | 32.92 | 122607 | 0.12 |
|  | *N. lugens* | 11285 | 34.40 | 43756 | 0.08 |
|  | *G. buenoi* | 10002 | 32.27 | 196158 | 0.41 |
|  | *C. lectularius* | 6505 | 34.82 | 140490 | 0.68 |
|  | *R. prolixus* | 7026 | 33.95 | 148115 | 0.44 |
|  | *P. guildinii* | 32 | 36.46 | 432 | 0.26 |
|  | *H. halys* | 11501 | 30.88 | 248834 | 0.42 |
|  | *O. fasciatus* | 10987 | 32.66 | 280591 | 0.04 |
|  | *D. citri* | 4857 | 38.06 | 282668 | 1.91 |
|  | *P. venusta* | 7018 | 35.21 | 105025 | 0.32 |
|  | *D. coccus* | 186 | 58.14 | 225 | 0.02 |
|  | *A. pisum* | 5417 | 29.76 | 184683 | 0.74 |
| Phasmatodea | *T. cristinae* | 10293 | 34.82 | 180207 | 0.43 |
| Orthoptera | *L. migratoria* | 57598 | 40.68 | 1509546 | 0.72 |
| Isoptera | *Z. nevadensis* | 4850 | 38.18 | 76081 | 0.38 |
| Blattodea | *B. germanica* | 20372 | 34.50 | 667792 | 0.99 |
