# Supplementary figures and images for: Large-scale analysis reveals that the genome features of simple sequence repeats are generally conserved at the family level in insects
Source: BMC Genomics. 2017 Nov 6;18:848. doi: 10.1186/s12864-017-4234-0 (PMC5674736; doi:10.1186/s12864-017-4234-0)

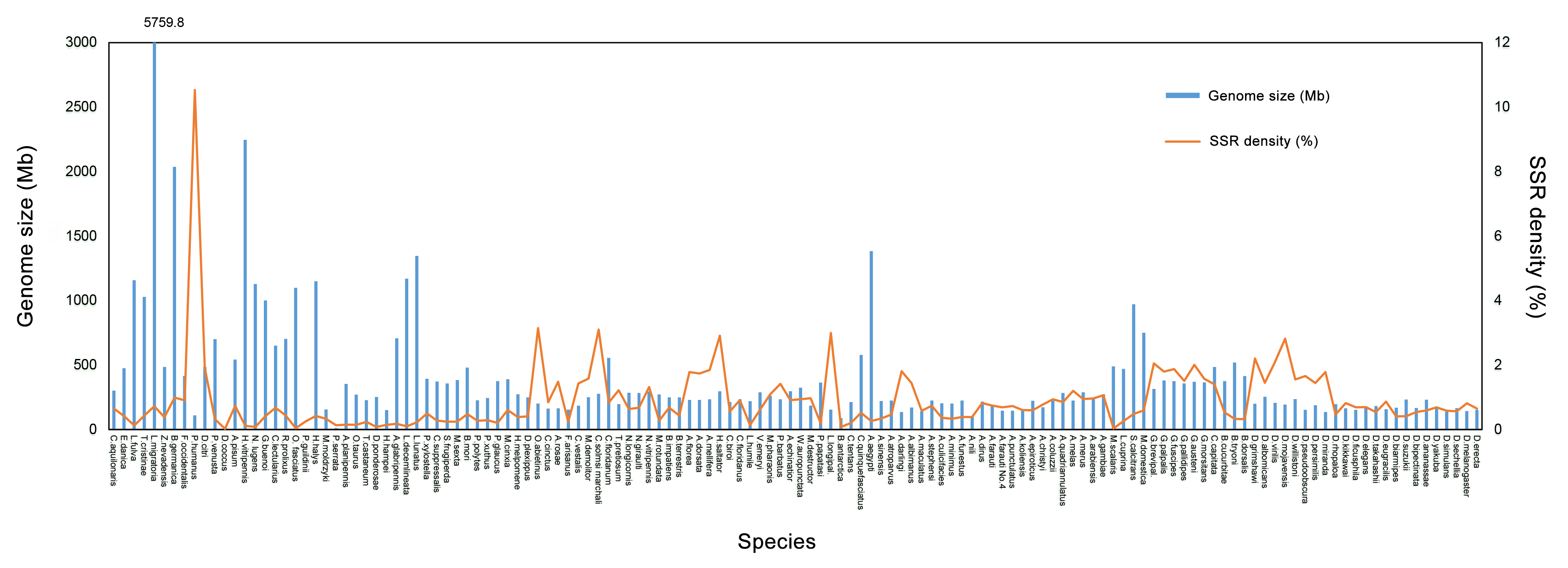

Supplement: Supplementary file 2 — Figure S1. The insect genome sizes and SSR densities of 136 insects, showing that SSR densities have no relationship with genome size. (TIFF 2488 kb) [file 12864_2017_4234_MOESM2_ESM.tif]

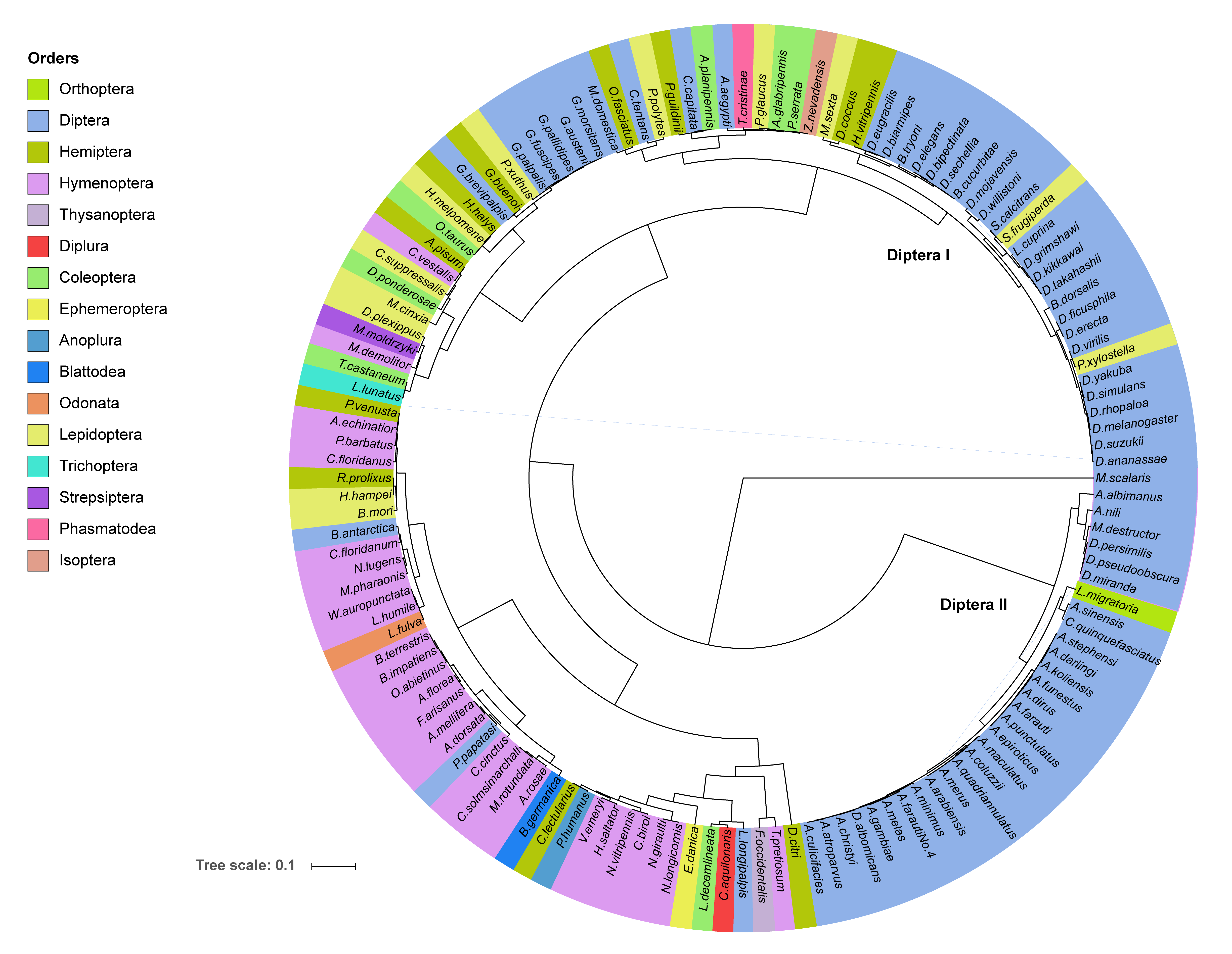

Supplement: Supplementary file 10 — Figure S2. Phylogenetic analysis of 136 insect genomes using the relative abundance information from di-nucleotide SSRs. (TIFF 2484 kb) [file 12864_2017_4234_MOESM10_ESM.tif]

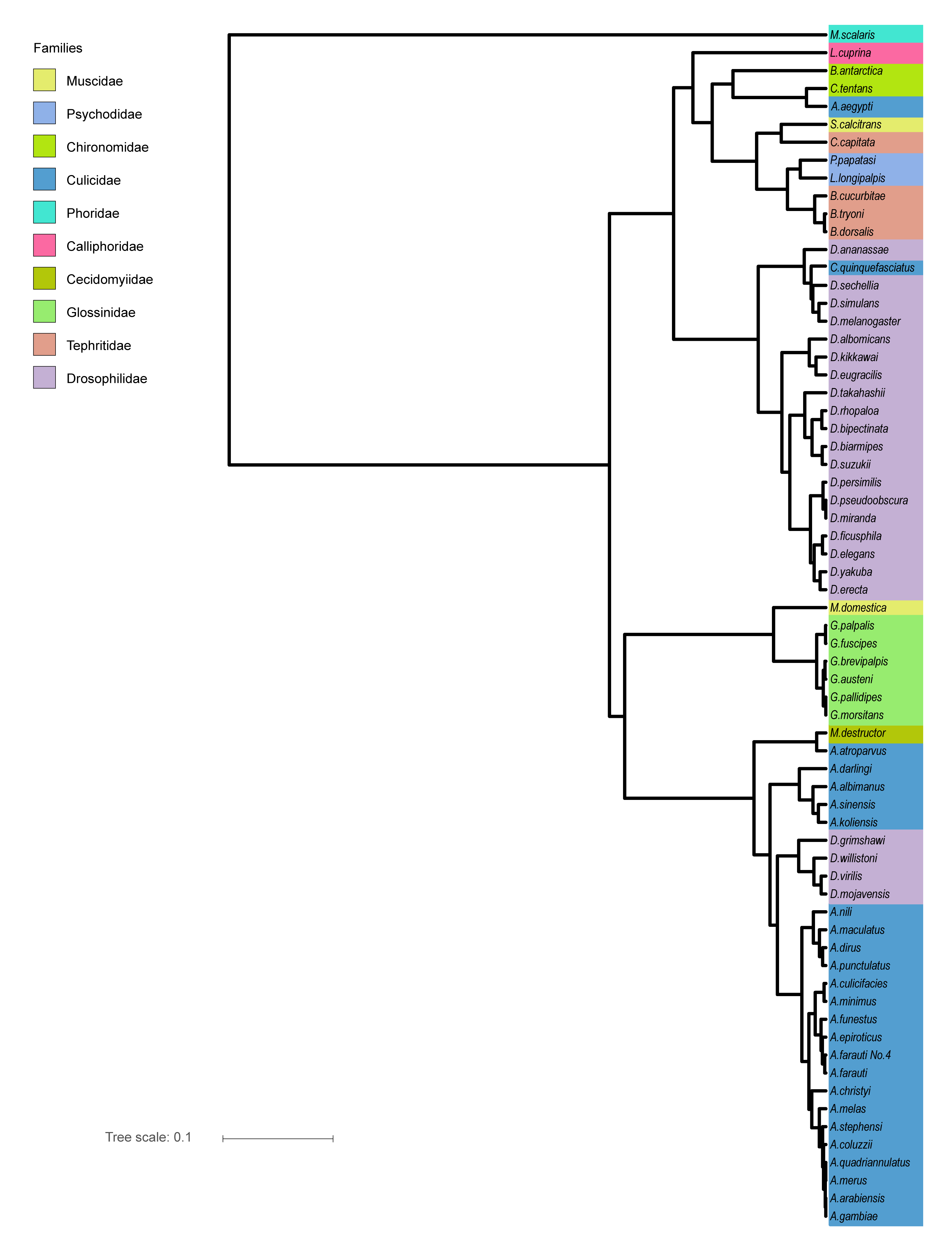

Supplement: Supplementary file 11 — Figure S3. Phylogenetic analysis of Diptera using the relative abundance information from six types of SSRs, showing that dipteran insects can be clearly classified at the family level. (TIFF 1521 kb) [file 12864_2017_4234_MOESM11_ESM.tif]

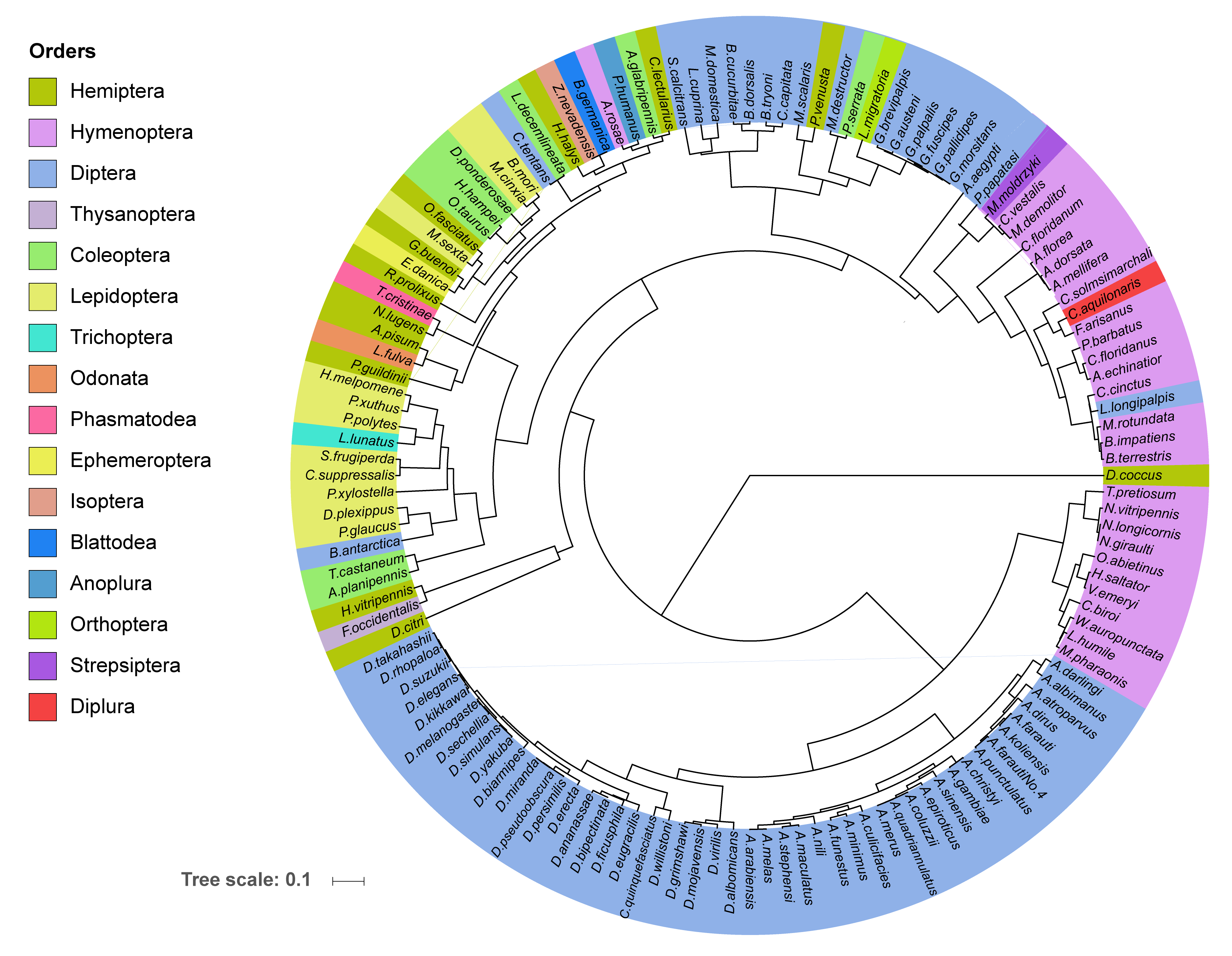

Supplement: Supplementary file 12 — Figure S4. Phylogenetic analysis of 136 insect genomes using the relative abundance information from tri-nucleotide SSRs (TIFF 1694 kb) [file 12864_2017_4234_MOESM12_ESM.tif]

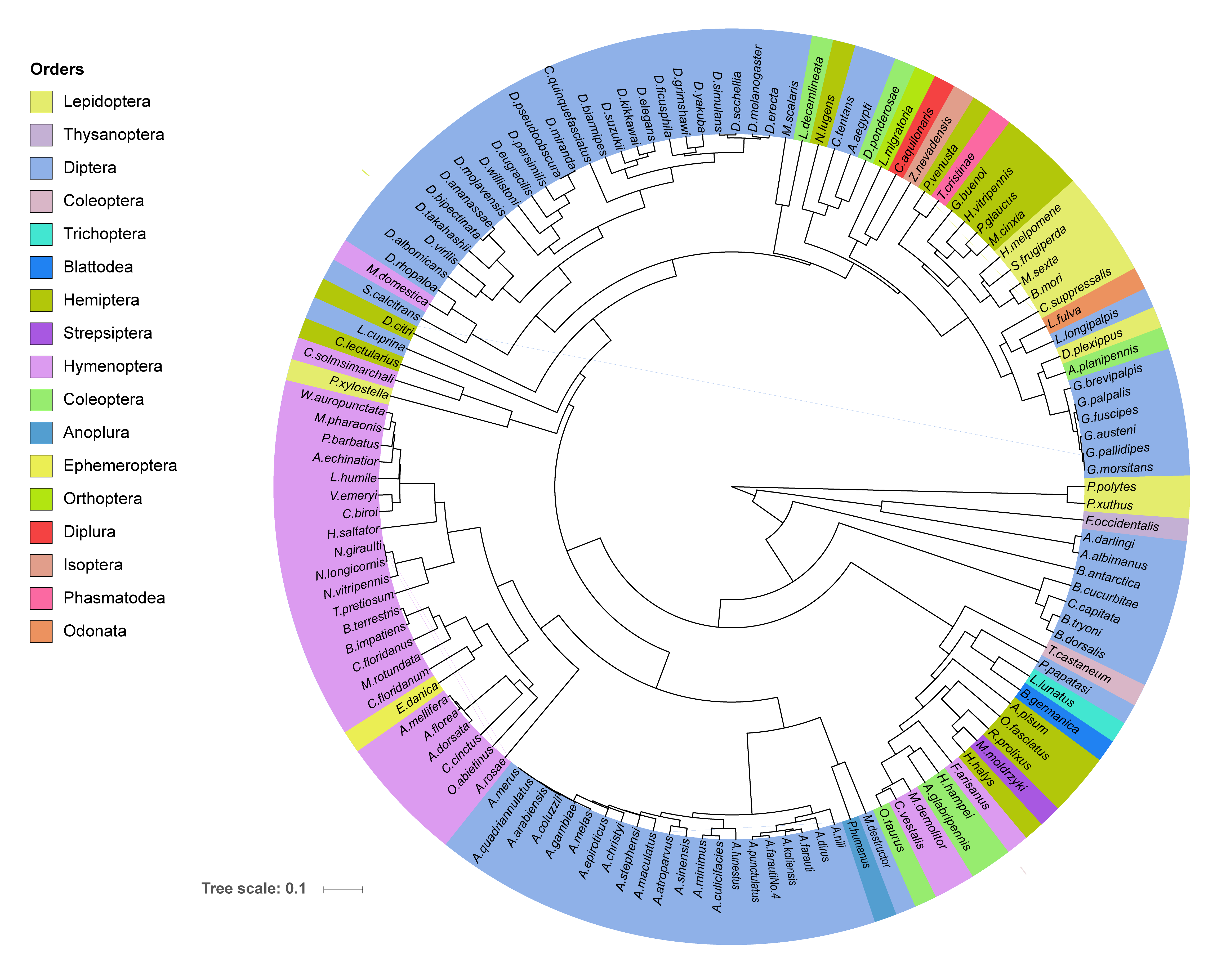

Supplement: Supplementary file 13 — Figure S5. Phylogenetic analysis of 136 insects using the relative abundance information from tetra-nucleotide SSR. (TIFF 2340 kb) [file 12864_2017_4234_MOESM13_ESM.tif]

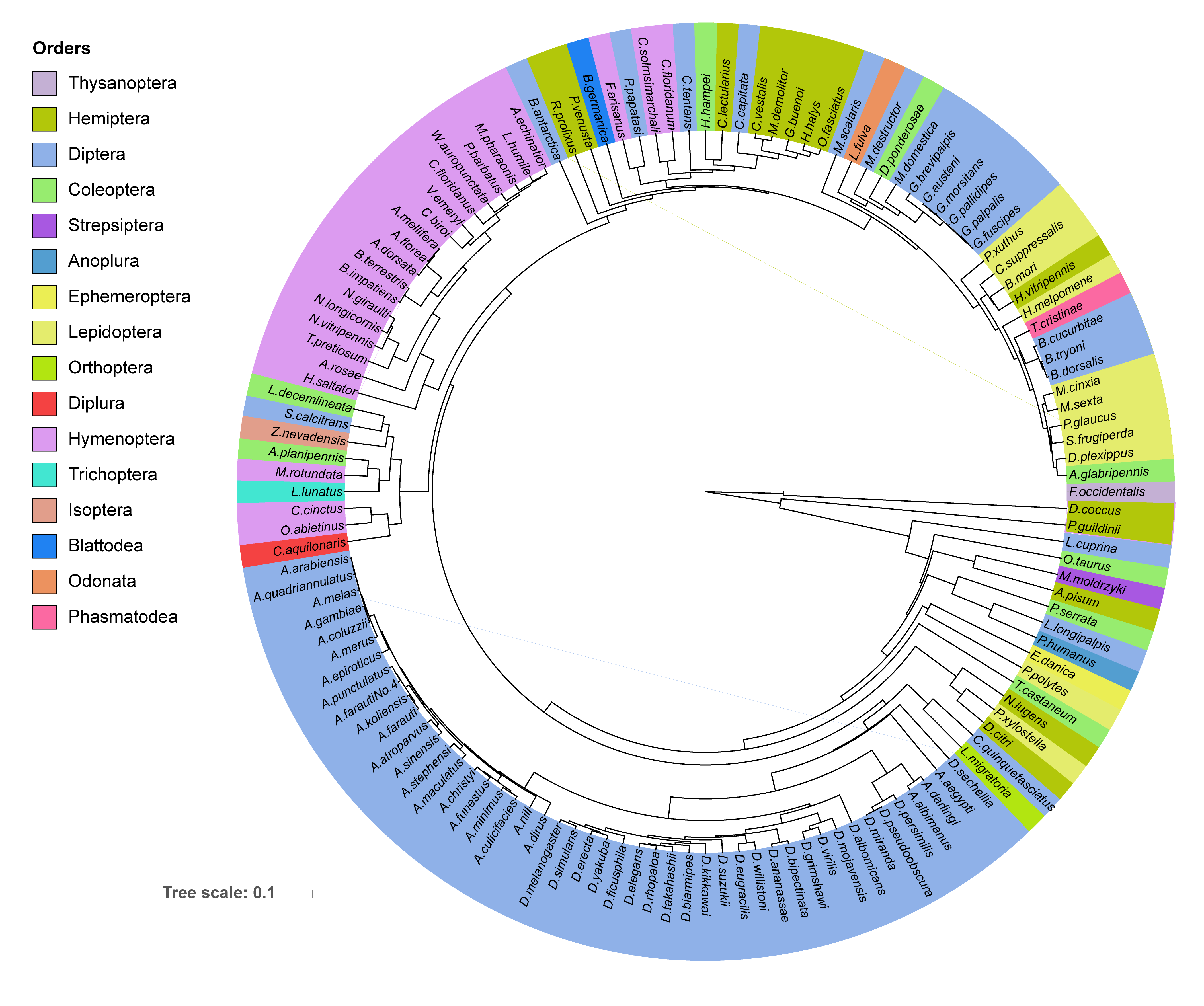

Supplement: Supplementary file 14 — Figure S6. Phylogenetic analysis of 136 insects using the relative abundance information from penta-nucleotide SSR. (TIFF 2093 kb) [file 12864_2017_4234_MOESM14_ESM.tif]
