# Supplementary material for: Large-scale analysis reveals that the genome features of simple sequence repeats are generally conserved at the family level in insects
Source: BMC Genomics. 2017 Nov 6;18:848. doi: 10.1186/s12864-017-4234-0 (PMC5674736; doi:10.1186/s12864-017-4234-0)
Supplement: Supplementary file 3 — Table S2. Perfect and imperfect SSRs in insect genomes. (DOCX 36 kb) [file 12864_2017_4234_MOESM3_ESM.docx]

**Table S2. Information of perfect and imperfect SSRs in insect Genomes**

| **Species** | **Average length of perfect SSR (bp)** | **Average length of imperfect SSR (bp** | **No. SSR <=30 bp with >= 3 mismatches** | **No. SSR >= 30 bp with < 3 mismatches** | **Imperfect-SSR percentage**  **(%)** | **Mismatch percentage**  **(%)** |
| --- | --- | --- | --- | --- | --- | --- |
| *C.aquilonaris* | 26 | 41 | 2543 | 4758 | 19.86 | 1.33 |
| *E.danica* | 25 | 37 | 1128 | 4900 | 16.51 | 0.87 |
| *L.fulva* | 17 | 28 | 149 | 106 | 15.06 | 2.32 |
| *M.moldrzyki* | 21 | 37 | 2835 | 4180 | 18.07 | 1.48 |
| *O.abietinus* | 18 | 30 | 456 | 412 | 24.51 | 1.29 |
| *C.cinctus* | 26 | 40 | 1646 | 4312 | 24.59 | 1.57 |
| *A.rosae* | 21 | 32 | 3768 | 8329 | 26.58 | 1.80 |
| *C.vestalis* | 24 | 40 | 4143 | 5525 | 22.93 | 1.57 |
| *M.demolitor* | 27 | 41 | 747 | 975 | 25.66 | 1.62 |
| *F.arisanus* | 19 | 33 | 4212 | 5072 | 26.17 | 2.19 |
| *H.saltator* | 24 | 46 | 2233 | 3710 | 38.73 | 2.56 |
| *C.biroi* | 21 | 32 | 6561 | 8295 | 28.06 | 1.95 |
| *C.floridanus* | 22 | 38 | 3985 | 5432 | 35.61 | 2.67 |
| *L.humile* | 22 | 39 | 1999 | 6351 | 33.85 | 1.31 |
| *V.emeryi* | 23 | 36 | 5152 | 10998 | 30.80 | 2.05 |
| *M.pharaonis* | 25 | 37 | 1547 | 3448 | 30.53 | 1.82 |
| *P.barbatus* | 27 | 44 | 6298 | 13483 | 30.51 | 1.81 |
| *A.echinatior* | 24 | 38 | 3662 | 6894 | 33.44 | 2.14 |
| *W.auropunctata* | 23 | 36 | 1966 | 5778 | 33.79 | 2.13 |
| *M.rotundata* | 25 | 43 | 3615 | 4958 | 27.79 | 2.10 |
| *B.impatiens* | 30 | 49 | 3032 | 4355 | 33.94 | 1.98 |
| *B.terrestris* | 21 | 36 | 7011 | 13110 | 33.20 | 2.48 |
| *A.florea* | 21 | 33 | 10205 | 13596 | 26.23 | 1.81 |
| *A.dorsata* | 20 | 34 | 10882 | 13837 | 31.31 | 2.44 |
| *A.mellifera* | 21 | 35 | 20600 | 35074 | 30.94 | 2.41 |
| *C.solmsi marchali* | 29 | 47 | 8732 | 10585 | 31.72 | 2.12 |
| *C.floridanum* | 21 | 35 | 1415 | 1381 | 21.46 | 1.78 |
| *T.pretiosum* | 20 | 29 | 1323 | 6713 | 23.09 | 1.44 |
| *N.longicornis* | 23 | 34 | 5439 | 9519 | 20.54 | 1.14 |
| *N.giraulti* | 24 | 36 | 1327 | 6094 | 20.84 | 1.11 |
| *N.vitripennis* | 30 | 43 | 1620 | 1600 | 23.41 | 1.08 |
| *M.scalaris* | 16 | 22 | 21481 | 33131 | 6.91 | 0.43 |
| *G.brevipal.* | 21 | 37 | 15324 | 24273 | 35.21 | 2.92 |
| *G.palpalis* | 22 | 35 | 21027 | 23739 | 33.16 | 2.29 |
| *G.fuscipes* | 22 | 35 | 15177 | 25753 | 33.00 | 2.27 |
| *G.pallidipes* | 21 | 36 | 4670 | 6275 | 36.73 | 2.66 |
| *G.austeni* | 22 | 38 | 16430 | 31550 | 35.74 | 2.65 |
| *G.morsitans* | 21 | 36 | 43208 | 135397 | 35.41 | 2.52 |
| *L.cuprina* | 25 | 73 | 678 | 1264 | 36.17 | 1.32 |
| *S,calcitrans* | 18 | 45 | 4914 | 12783 | 27.99 | 3.15 |
| *M.domestica* | 19 | 30 | 5786 | 7661 | 22.49 | 1.63 |
| *D.albomicans* | 18 | 27 | 14036 | 18582 | 30.67 | 2.17 |
| *D.virilis* | 21 | 42 | 19127 | 24165 | 40.96 | 3.23 |
| *D.mojavensis* | 21 | 37 | 17099 | 22804 | 40.49 | 3.01 |
| *D.grimshawi* | 20 | 35 | 7229 | 13573 | 43.80 | 3.20 |
| *D.willistoni* | 19 | 31 | 4648 | 9830 | 33.31 | 2.33 |
| *D.pseudoobscura* | 19 | 31 | 5972 | 10437 | 30.10 | 2.14 |
| *D.persimilis* | 20 | 32 | 4164 | 9643 | 29.31 | 2.19 |
| *D.miranda* | 20 | 31 | 3057 | 2544 | 29.67 | 2.06 |
| *D.rhopaloa* | 18 | 34 | 2985 | 2811 | 30.67 | 2.75 |
| *D.kikkawai* | 18 | 32 | 2809 | 3181 | 26.00 | 2.22 |
| *D.ficusphila* | 19 | 31 | 2412 | 3844 | 30.57 | 2.46 |
| *D.elegans* | 19 | 33 | 3017 | 2339 | 28.08 | 2.13 |
| *D.takahashii* | 19 | 37 | 2692 | 3953 | 28.16 | 2.50 |
| *D.eugracilis* | 19 | 39 | 1477 | 1509 | 30.37 | 2.68 |
| *D.biarmipes* | 19 | 36 | 1440 | 1498 | 25.33 | 2.26 |
| *D.suzukii* | 19 | 43 | 2991 | 3476 | 23.01 | 1.81 |
| *D.yakuba* | 19 | 34 | 1696 | 1817 | 30.01 | 2.54 |
| *D.simulans* | 18 | 32 | 1793 | 1635 | 26.62 | 2.22 |
| *D.sechellia* | 18 | 39 | 2528 | 3248 | 23.93 | 2.16 |
| *D.melanogaster* | 19 | 35 | 2411 | 3262 | 29.67 | 2.47 |
| *D.erecta* | 20 | 37 | 2003 | 2092 | 30.63 | 2.35 |
| *D.bipectinata* | 18 | 31 | 2700 | 3243 | 26.94 | 2.21 |
| *D.ananassae* | 19 | 36 | 2872 | 3182 | 25.97 | 2.43 |
| *B.cucurbitae* | 18 | 33 | 3312 | 3034 | 22.51 | 1.80 |
| *B.tryoni* | 17 | 32 | 2149 | 1786 | 24.33 | 2.11 |
| *B.dorsalis* | 17 | 29 | 21571 | 20432 | 22.70 | 1.92 |
| *C.capitata* | 22 | 42 | 718 | 1346 | 36.28 | 2.86 |
| *M.destructor* | 20 | 30 | 14896 | 33089 | 26.51 | 1.69 |
| *P.papatasi* | 19 | 30 | 13916 | 9983 | 19.28 | 1.47 |
| *L. longipal.* | 17 | 29 | 860 | 1496 | 16.52 | 3.21 |
| *A.sinensis* | 18 | 29 | 1786 | 3058 | 23.36 | 1.67 |
| *A.atroparvus* | 19 | 33 | 3404 | 7236 | 25.98 | 1.94 |
| *A.darlingi* | 19 | 30 | 3258 | 8448 | 28.86 | 1.91 |
| *A.albimanus* | 20 | 31 | 716 | 1242 | 28.70 | 1.84 |
| *A.culicifacies* | 18 | 28 | 723 | 1414 | 19.75 | 1.43 |
| *A.minimus* | 19 | 30 | 1113 | 2127 | 22.45 | 1.56 |
| *A.funestus* | 19 | 30 | 825 | 1690 | 24.88 | 1.76 |
| *A.maculatus* | 19 | 29 | 1624 | 4145 | 22.58 | 1.57 |
| *A.stephensi* | 19 | 30 | 418 | 1072 | 22.21 | 1.48 |
| *A.nili* | 19 | 29 | 2025 | 4931 | 25.65 | 1.72 |
| *A.farauti* | 20 | 32 | 1426 | 2947 | 28.73 | 1.88 |
| *A.farauti No.4* | 20 | 31 | 1777 | 3539 | 27.37 | 1.84 |
| *A.punctulatus* | 19 | 30 | 1121 | 1651 | 30.35 | 2.09 |
| *A.koliensis* | 18 | 28 | 2531 | 5963 | 25.18 | 1.75 |
| *A.dirus* | 21 | 33 | 1737 | 4317 | 27.87 | 1.79 |
| *A.epiroticus* | 20 | 32 | 1491 | 4099 | 26.02 | 1.75 |
| *A.christyi* | 20 | 31 | 3569 | 5612 | 24.23 | 1.59 |
| *A.coluzzii* | 20 | 36 | 3463 | 6793 | 27.34 | 1.96 |
| *A.quadriannulatus* | 21 | 34 | 3434 | 8451 | 25.97 | 1.75 |
| *A.melas* | 21 | 34 | 3987 | 8111 | 25.17 | 1.61 |
| *A.merus* | 22 | 36 | 3484 | 6630 | 26.78 | 1.73 |
| *A.arabiensis* | 21 | 35 | 5193 | 7799 | 26.06 | 1.77 |
| *A.gambiae* | 21 | 42 | 4686 | 3876 | 28.17 | 1.98 |
| *C.quinquefasciatus* | 21 | 57 | 3262 | 4865 | 19.47 | 2.28 |
| *A.aegypti* | 21 | 53 | 17 | 48 | 15.31 | 1.78 |
| *B.antarctica* | 18 | 27 | 279 | 491 | 9.50 | 0.67 |
| *C.tentans* | 17 | 27 | 1309 | 2053 | 15.80 | 1.17 |
| *L.lunatus* | 18 | 31 | 3 | 9 | 18.63 | 1.56 |
| *P.xylostella* | 20 | 34 | 857 | 1142 | 16.12 | 1.13 |
| *C.suppressalis* | 17 | 27 | 654 | 685 | 16.03 | 1.25 |
| *P.polytes* | 18 | 32 | 467 | 1266 | 15.58 | 1.14 |
| *P.xuthus* | 19 | 39 | 4209 | 2358 | 16.01 | 1.68 |
| *P.glaucus* | 17 | 28 | 4 | 0 | 14.70 | 1.25 |
| *M.cinxia* | 22 | 38 | 26036 | 49609 | 22.15 | 1.55 |
| *H.melpomene* | 20 | 31 | 1368 | 2071 | 17.66 | 1.27 |
| *D.plexippus* | 22 | 35 | 3894 | 5540 | 22.79 | 1.57 |
| *S.frugiperda* | 18 | 30 | 291 | 554 | 16.20 | 1.24 |
| *M.sexta* | 19 | 30 | 2581 | 4163 | 17.26 | 1.24 |
| *B.mori* | 19 | 33 | 6885 | 14042 | 18.74 | 1.44 |
| *P.serrata* | 17 | 26 | 300 | 637 | 14.20 | 1.13 |
| *A.planipennis* | 18 | 29 | 6868 | 12874 | 14.25 | 1.00 |
| *O.taurus* | 17 | 27 | 193 | 257 | 15.25 | 1.26 |
| *D.ponderosae* | 17 | 28 | 2138 | 4320 | 17.68 | 1.36 |
| *H.hampei* | 17 | 24 | 1772 | 2705 | 16.60 | 1.19 |
| *A.glabripennis* | 20 | 35 | 1132 | 1069 | 20.93 | 1.67 |
| *L.decemlineata* | 18 | 33 | 6245 | 10635 | 16.60 | 1.26 |
| *T.castaneum* | 21 | 52 | 800 | 893 | 28.80 | 2.36 |
| *F.occidentalis* | 23 | 39 | 923 | 994 | 29.78 | 2.15 |
| *P.humanus* | 21 | 33 | 1034 | 1709 | 22.19 | 1.53 |
| *H.vitripennis* | 19 | 32 | 3070 | 14563 | 14.31 | 1.05 |
| *N.lugens* | 21 | 29 | 9118 | 12751 | 8.36 | 0.63 |
| *G.buenoi* | 19 | 29 | 15871 | 16953 | 18.45 | 1.39 |
| *C.lectularius* | 24 | 45 | 9 | 11 | 35.13 | 2.75 |
| *R.prolixus* | 19 | 31 | 834 | 1305 | 18.61 | 1.49 |
| *P.guildinii* | 18 | 27 | 16504 | 30738 | 13.66 | 0.97 |
| *H.halys* | 18 | 27 | 183 | 293 | 15.74 | 1.18 |
| *O. fasciatus* | 20 | 34 | 31133 | 27406 | 17.92 | 1.91 |
| *D.citri* | 25 | 48 | 733 | 899 | 32.26 | 2.82 |
| *P.venusta* | 19 | 35 | 1 | 1 | 14.02 | 1.60 |
| *D.coccus* | 17 | 24 | 6441 | 9631 | 14.67 | 1.01 |
| *A.pisum* | 19 | 31 | 14354 | 9316 | 24.13 | 1.82 |
| *T.cristinae* | 21 | 36 | 3748 | 6189 | 20.20 | 1.59 |
| *L.migratoria* | 25 | 38 | 6518 | 14668 | 20.19 | 1.26 |
| *Z.nevadensis* | 20 | 45 | 38729 | 71519 | 17.18 | 1.93 |
| *B.germanica* | 26 | 43 | 3151 | 3215 | 25.74 | 1.81 |
