# Supplementary material for: Large-scale analysis reveals that the genome features of simple sequence repeats are generally conserved at the family level in insects
Source: BMC Genomics. 2017 Nov 6;18:848. doi: 10.1186/s12864-017-4234-0 (PMC5674736; doi:10.1186/s12864-017-4234-0)
Supplement: Supplementary file 4 — Table S3. Percentage (%) of different types of SSRs. (DOCX 14 kb) [file 12864_2017_4234_MOESM4_ESM.docx]

**Table S3. Percentage (%) of different types of SSRs**

| SSR classes | Mean±SE* | Range |
| --- | --- | --- |
| Mono-nucleotide | 11.6±1.0c | 0.0−53.8 |
| Di-nucleotide | 27.2±1.4a | 0.4−71.5 |
| Tri-nucleotide | 22.0±0.7a | 5.6−42.7 |
| Tetra-nucleotide | 17.9±0.8b | 4.8−47.5 |
| Penta-nucleotide | 14.6±0.7b | 2.5−39.9 |
| Hexa-nucleotide | 6.7±0.5d | 0.6−26.3 |

*: Means following by same lowercase letters were not significantly different with each other.
