# Supplementary material for: Large-scale analysis reveals that the genome features of simple sequence repeats are generally conserved at the family level in insects
Source: BMC Genomics. 2017 Nov 6;18:848. doi: 10.1186/s12864-017-4234-0 (PMC5674736; doi:10.1186/s12864-017-4234-0)
Supplement: Supplementary file 5 — Table S4. Percentage of different types of SSRs in all SSRs (DOCX 15 kb) [file 12864_2017_4234_MOESM5_ESM.docx]

**Table S4. Percentage of different types of SSRs in all SSRs**

| SSR classes | No. | Motifs | Mean±SE* | Range |
| --- | --- | --- | --- | --- |
| Mono-nucleotide | A01 | A\|T | 10.3±1.0a | 0.0−52.0 |
|  | A02 | G\|C | 1.2±0.2d | 0.0−22.6 |
|  |  |  |  |  |
| Di-nucleotide | B01 | AT\|TA | 6.6±0.5b | 0.1−29.6 |
|  | B02 | AG\|GA\|CT\|TC | 10.2±0.9a | 0.1−56.8 |
|  | B03 | AC\|CA\|GT\|TG | 10.0±0.8a | 0.0−35.8 |
|  | B04 | GC\|CG | 0.4±0.1e | 0.0−6.1 |
|  |  |  |  |  |
| Tri-nucleotide | C01 | AAT\|ATA\|TAA\|TAT\|ATT\|TTA | 6.3±0.6b | 0.3−33.7 |
|  | C02 | GGC\|GCG\|CGG\|CGC\|GCC\|CCG | 0.9±0.2de | 0.0−30.2 |
|  | C03 | AAG\|AGA\|GAA\|TCT\|CTT\|TTC | 2.0±0.2cd | 0.0−13.5 |
|  | C04 | AAC\|ACA\|CAA\|TGT\|GTT\|TTG | 2.7±0.2c | 0.2−16.1 |
|  | C05 | AGT\|GTA\|TAG\|CAT\|ATC\|TCA | 1.6±0.1cd | 0.0−9.4 |
|  | C06 | ACT\|CTA\|TAC\|GAT\|ATG\|TGA | 1.5±0.1cd | 0.3−8.7 |
|  | C07 | AGC\|GCA\|CAG\|CGT\|GTC\|TCG | 2.6±0.2c | 0.0−13.6 |
|  | C08 | ACG\|CGA\|GAC\|GCT\|CTG\|TGC | 2.6±0.2c | 0.0−13.8 |
|  | C09 | AGG\|GGA\|GAG\|CCT\|CTC\|TCC | 0.9±0.1d | 0.0−6.4 |
|  | C10 | ACC\|CCA\|CAC\|GGT\|GTG\|TGG | 1.0±0.1d | 0.0−6.5 |

*: Means following by same lowercase letters were not significantly different with each other;

SSR motifs accounting for lower pencentage were indicated with red colour
