# Supplementary material for: Large-scale analysis reveals that the genome features of simple sequence repeats are generally conserved at the family level in insects
Source: BMC Genomics. 2017 Nov 6;18:848. doi: 10.1186/s12864-017-4234-0 (PMC5674736; doi:10.1186/s12864-017-4234-0)
Supplement: Supplementary file 6 — Table S5. Percentage of different types of SSRs calculated within classes. (DOCX 15 kb) [file 12864_2017_4234_MOESM6_ESM.docx]

**Table S5.** **Percentage of different types of SSRs calculated within its classes**

| SSR classes | No. | Motifs | Mean±SE* | Range |
| --- | --- | --- | --- | --- |
| Mono-nucleotide | A01 | A\|T | 82.4±1.9 |  |
|  | A02 | G\|C | 17.6±1.9 |  |
|  |  |  |  |  |
| Di-nucleotide | B01 | AT\|TA | 30.5±1.9b | 0.3−83.5 |
|  | B02 | AG\|GA\|CT\|TC | 32.2±1.6ab | 2.6−93.9 |
|  | B03 | AC\|CA\|GT\|TG | 36.4±1.9a | 0.0−77.5 |
|  | B04 | GC\|CG | 0.9±0.2c | 0.0−15.4 |
|  |  |  |  |  |
| Tri-nucleotide | C01 | AAT\|ATA\|TAA\|TAT\|ATT\|TTA | 30.0±2.1a | 0.6−85.3 |
|  | C02 | GGC\|GCG\|CGG\|CGC\|GCC\|CCG | 3.8±0.6d | 0.0−70.8 |
|  | C03 | AAG\|AGA\|GAA\|TCT\|CTT\|TTC | 9.4±0.7bc | 0.0−49.3 |
|  | C04 | AAC\|ACA\|CAA\|TGT\|GTT\|TTG | 12.7±1.1b | 1.0−66.4 |
|  | C05 | AGT\|GTA\|TAG\|CAT\|ATC\|TCA | 6.9±0.5c | 0.0−36.2 |
|  | C06 | ACT\|CTA\|TAC\|GAT\|ATG\|TGA | 6.9±0.5c | 1.0−31.1 |
|  | C07 | AGC\|GCA\|CAG\|CGT\|GTC\|TCG | 10.9±0.8bc | 0.0−34.1 |
|  | C08 | ACG\|CGA\|GAC\|GCT\|CTG\|TGC | 10.9±0.8bc | 0.1−34.7 |
|  | C09 | AGG\|GGA\|GAG\|CCT\|CTC\|TCC | 4.2±0.4d | 0.1−21.5 |
|  | C10 | ACC\|CCA\|CAC\|GGT\|GTG\|TGG | 4.3±0.3d | 0.0−19.6 |

*: Means following by same lowercase letters were not significantly different with each other；SSR motifs accounting for lower pencentage were indicated with red colour
