# Supplementary material for: Large-scale analysis reveals that the genome features of simple sequence repeats are generally conserved at the family level in insects
Source: BMC Genomics. 2017 Nov 6;18:848. doi: 10.1186/s12864-017-4234-0 (PMC5674736; doi:10.1186/s12864-017-4234-0)
Supplement: Supplementary file 7 — Table S6. The sequences of SSR motifs. (DOCX 21 kb) [file 12864_2017_4234_MOESM7_ESM.docx]

**Table S6. SSR motifs**

| SSR classes | No. | Motifs |
| --- | --- | --- |
| Mono-nucleotide | A01 | A\|T |
|  | A02 | G\|C |
| Di-nucleotide | B01 | AT\|TA |
|  | B02 | AG\|GA\|CT\|TC |
|  | B03 | AC\|CA\|GT\|TG |
| Tri-nucleotide | C01 | AAT\|ATA\|TAA\|TAT\|ATT\|TTA |
|  | C02 | GGC\|GCG\|CGG\|CGC\|GCC\|CCG |
|  | C03 | AAG\|AGA\|GAA\|TCT\|CTT\|TTC |
|  | C04 | AAC\|ACA\|CAA\|TGT\|GTT\|TTG |
|  | C05 | AGT\|GTA\|TAG\|CAT\|ATC\|TCA |
|  | C06 | ACT\|CTA\|TAC\|GAT\|ATG\|TGA |
|  | C07 | AGC\|GCA\|CAG\|CGT\|GTC\|TCG |
|  | C08 | ACG\|CGA\|GAC\|GCT\|CTG\|TGC |
| Tetra-nucleotide | D01 | AAAT\|AATA\|ATAA\|TAAA\|TTAT\|TATT\|ATTT\|TTTA |
|  | D02 | AAAC\|AACA\|ACAA\|CAAA\|TTGT\|TGTT\|GTTT\|TTTG |
|  | D03 | AAAG\|AAGA\|AGAA\|GAAA\|TTCT\|TCTT\|CTTT\|TTTC |
| Penta-nucleotide | E01 | AAAAT\|AAATA\|AATAA\|ATAAA\|TAAAA\|TTTAT\|TTATT\|TATTT\|ATTTT\|TTTTA |
|  | E02 | AATCC\|ATCCA\|TCCAA\|CCAAT\|CAATC\|TAGGT\|AGGTT\|GGTTA\|GTTAG\|TTAGG |
|  | E03 | AACCC\|ACCCA\|CCCAA\|CCAAC\|CAACC\|TGGGT\|GGGTT\|GGTTG\|GTTGG\|TTGGG |
|  | E04 | AAAAC\|AAACA\|AACAA\|ACAAA\|CAAAA\|TTTGT\|TTGTT\|TGTTT\|GTTTT\|TTTTG |
|  | E05 | GGGGC\|GGGCG\|GGCGG\|GCGGG\|CGGGG\|CCCGC\|CCGCC\|CGCCC\|GCCCC\|CCCCG |
|  | E06 | AATAG\|ATAGA\|TAGAA\|AGAAT\|GAATA\|TATCT\|ATCTT\|TCTTA\|CTTAT\|TTATC |
|  | E07 | GGCGC\|GCGCG\|CGCGG\|GCGGC\|CGGCG\|CGCGC\|GCGCC\|CGCCG\|GCCGC\|CCGCG |
|  | E08 | AATAT\|ATATA\|TATAA\|ATAAT\|TAATA\|TATAT\|ATATT\|TATTA\|ATTAT\|TTATA |
|  | E09 | AAATC\|AATCA\|ATCAA\|TCAAA\|CAAAT\|TTAGT\|TAGTT\|AGTTT\|GTTTA\|TTTAG |
|  | E10 | AACCT\|ACCTA\|CCTAA\|CTAAC\|TAACC\|TGGAT\|GGATT\|GATTG\|ATTGG\|TTGGA |
|  | E11 | AAAAG\|AAAGA\|AAGAA\|AGAAA\|GAAAA\|TTTCT\|TTCTT\|TCTTT\|CTTTT\|TTTTC |
|  | E12 | AAATT\|AATTA\|ATTAA\|TTAAA\|TAAAT\|TTAAT\|TAATT\|AATTT\|ATTTA\|TTTAA |
|  | E13 | AATCT\|ATCTA\|TCTAA\|CTAAT\|TAATC\|TAGAT\|AGATT\|GATTA\|ATTAG\|TTAGA |
|  | E14 | AGACT\|GACTA\|ACTAG\|CTAGA\|TAGAC\|CTGAT\|TGATC\|GATCT\|ATCTG\|TCTGA |
|  | E15 | AAGAC\|AGACA\|GACAA\|ACAAG\|CAAGA\|TCTGT\|CTGTT\|TGTTC\|GTTCT\|TTCTG |
| Hexa-nucleotide | F01 | AAAAAT\|AAAATA\|AAATAA\|AATAAA\|ATAAAA\|TAAAAA\|TTTTAT\|TTTATT\|TTATTT\|TATTTT\|ATTTTT\|TTTTTA |
|  | F02 | AGATGT\|GATGTA\|ATGTAG\|TGTAGA\|GTAGAT\|TAGATG\|CTACAT\|TACATC\|ACATCT\|CATCTA\|ATCTAC\|TCTACA |
|  | F03 | AGCGTC\|GCGTCA\|CGTCAG\|GTCAGC\|TCAGCG\|CAGCGT\|CGCAGT\|GCAGTC\|CAGTCG\|AGTCGC\|GTCGCA\|TCGCAG |
|  | F04 | AGAGGG\|GAGGGA\|AGGGAG\|GGGAGA\|GGAGAG\|GAGAGG\|CTCCCT\|TCCCTC\|CCCTCT\|CCTCTC\|CTCTCC\|TCTCCC |
|  | F05 | GGGCGC\|GGCGCG\|GCGCGG\|CGCGGG\|GCGGGC\|CGGGCG\|CCGCGC\|CGCGCC\|GCGCCC\|CGCCCG\|GCCCGC\|CCCGCG |
|  | F06 | AAATTC\|AATTCA\|ATTCAA\|TTCAAA\|TCAAAT\|CAAATT\|TTAAGT\|TAAGTT\|AAGTTT\|AGTTTA\|GTTTAA\|TTTAAG |
|  | F07 | AAGAAC\|AGAACA\|GAACAA\|AACAAG\|ACAAGA\|CAAGAA\|TCTTGT\|CTTGTT\|TTGTTC\|TGTTCT\|GTTCTT\|TTCTTG |
|  | F08 | AAACCG\|AACCGA\|ACCGAA\|CCGAAA\|CGAAAC\|GAAACC\|TTGGCT\|TGGCTT\|GGCTTT\|GCTTTG\|CTTTGG\|TTTGGC |
|  | F09 | AAGACC\|AGACCA\|GACCAA\|ACCAAG\|CCAAGA\|CAAGAC\|TCTGGT\|CTGGTT\|TGGTTC\|GGTTCT\|GTTCTG\|TTCTGG |
|  | F10 | ACATAT\|CATATA\|ATATAC\|TATACA\|ATACAT\|TACATA\|GTATAT\|TATATG\|ATATGT\|TATGTA\|ATGTAT\|TGTATA |
|  | F11 | AGATAC\|GATACA\|ATACAG\|TACAGA\|ACAGAT\|CAGATA\|CTATGT\|TATGTC\|ATGTCT\|TGTCTA\|GTCTAT\|TCTATG |
|  | F12 | AATGCG\|ATGCGA\|TGCGAA\|GCGAAT\|CGAATG\|GAATGC\|TACGCT\|ACGCTT\|CGCTTA\|GCTTAC\|CTTACG\|TTACGC |
|  | F13 | AAACTG\|AACTGA\|ACTGAA\|CTGAAA\|TGAAAC\|GAAACT\|TTGACT\|TGACTT\|GACTTT\|ACTTTG\|CTTTGA\|TTTGAC |
|  | F14 | GGGGGC\|GGGGCG\|GGGCGG\|GGCGGG\|GCGGGG\|CGGGGG\|CCCCGC\|CCCGCC\|CCGCCC\|CGCCCC\|GCCCCC\|CCCCCG |
|  | F15 | AAAAAG\|AAAAGA\|AAAGAA\|AAGAAA\|AGAAAA\|GAAAAA\|TTTTCT\|TTTCTT\|TTCTTT\|TCTTTT\|CTTTTT\|TTTTTC |
|  | F16 | AAAAAC\|AAAACA\|AAACAA\|AACAAA\|ACAAAA\|CAAAAA\|TTTTGT\|TTTGTT\|TTGTTT\|TGTTTT\|GTTTTT\|TTTTTG |
|  | F17 | AATAGT\|ATAGTA\|TAGTAA\|AGTAAT\|GTAATA\|TAATAG\|TATCAT\|ATCATT\|TCATTA\|CATTAT\|ATTATC\|TTATCA |
|  | F18 | AAAGAG\|AAGAGA\|AGAGAA\|GAGAAA\|AGAAAG\|GAAAGA\|TTCTCT\|TCTCTT\|CTCTTT\|TCTTTC\|CTTTCT\|TTTCTC |
|  | F19 | AAATAT\|AATATA\|ATATAA\|TATAAA\|ATAAAT\|TAAATA\|TTATAT\|TATATT\|ATATTT\|TATTTA\|ATTTAT\|TTTATA |
|  | F20 | ACACGC\|CACGCA\|ACGCAC\|CGCACA\|GCACAC\|CACACG\|GTGCGT\|TGCGTG\|GCGTGT\|CGTGTG\|GTGTGC\|TGTGCG |
|  | F21 | AAATCG\|AATCGA\|ATCGAA\|TCGAAA\|CGAAAT\|GAAATC\|TTAGCT\|TAGCTT\|AGCTTT\|GCTTTA\|CTTTAG\|TTTAGC |
|  | F22 | AGCCTC\|GCCTCA\|CCTCAG\|CTCAGC\|TCAGCC\|CAGCCT\|CGGAGT\|GGAGTC\|GAGTCG\|AGTCGG\|GTCGGA\|TCGGAG |
|  | F23 | AACTCG\|ACTCGA\|CTCGAA\|TCGAAC\|CGAACT\|GAACTC\|TGAGCT\|GAGCTT\|AGCTTG\|GCTTGA\|CTTGAG\|TTGAGC |
|  | F24 | AACGAC\|ACGACA\|CGACAA\|GACAAC\|ACAACG\|CAACGA\|TGCTGT\|GCTGTT\|CTGTTG\|TGTTGC\|GTTGCT\|TTGCTG |
|  | F25 | ACGACC\|CGACCA\|GACCAC\|ACCACG\|CCACGA\|CACGAC\|GCTGGT\|CTGGTG\|TGGTGC\|GGTGCT\|GTGCTG\|TGCTGG |
|  | F26 | AACAGC\|ACAGCA\|CAGCAA\|AGCAAC\|GCAACA\|CAACAG\|TGTCGT\|GTCGTT\|TCGTTG\|CGTTGT\|GTTGTC\|TTGTCG |
|  | F27 | AGGGGG\|GGGGGA\|GGGGAG\|GGGAGG\|GGAGGG\|GAGGGG\|CCCCCT\|CCCCTC\|CCCTCC\|CCTCCC\|CTCCCC\|TCCCCC |
|  | F28 | AAGATC\|AGATCA\|GATCAA\|ATCAAG\|TCAAGA\|CAAGAT\|TCTAGT\|CTAGTT\|TAGTTC\|AGTTCT\|GTTCTA\|TTCTAG |
|  | F29 | ACGGTG\|CGGTGA\|GGTGAC\|GTGACG\|TGACGG\|GACGGT\|GCCACT\|CCACTG\|CACTGC\|ACTGCC\|CTGCCA\|TGCCAC |
|  | F30 | AGTGGC\|GTGGCA\|TGGCAG\|GGCAGT\|GCAGTG\|CAGTGG\|CACCGT\|ACCGTC\|CCGTCA\|CGTCAC\|GTCACC\|TCACCG |
|  | F31 | AGACAT\|GACATA\|ACATAG\|CATAGA\|ATAGAC\|TAGACA\|CTGTAT\|TGTATC\|GTATCT\|TATCTG\|ATCTGT\|TCTGTA |
