# Supplementary material for: Large-scale analysis reveals that the genome features of simple sequence repeats are generally conserved at the family level in insects
Source: BMC Genomics. 2017 Nov 6;18:848. doi: 10.1186/s12864-017-4234-0 (PMC5674736; doi:10.1186/s12864-017-4234-0)
Supplement: Supplementary file 8 — Table S7. Relative abundance of perfect SSRs in different genomic regions. (DOCX 24 kb) [file 12864_2017_4234_MOESM8_ESM.docx]

**Table S7. The numbers of perfect SSRs in different genome regions**

| **Order** | **Species** | **Exon** | **Intron** | **Intergenic regions** | **Spanning exon-intron** | **Spanning intergenic-genetic** |
| --- | --- | --- | --- | --- | --- | --- |
| Anoplura | *P. humanus* | 2835 | 66194 | 292036 | 270 | 243 |
| Coleoptera | *D. ponderosae* | 211 | 1451 | 5101 | 15 | 9 |
|  | *T. castaneum* | 511 | 5538 | 6662 | 27 | 13 |
| Diptera | *A. aegypti* | 3769 | 230750 | 77267 | 388 | 36 |
|  | *A. coluzzii* | 2553 | 16504 | 38422 | 83 | 67 |
|  | *A. darlingi* | 19544 | 32849 | 151845 | 1699 | 350 |
|  | *A. gambiae* | 3072 | 21086 | 53164 | 60 | 67 |
|  | *A. sinensis* | 2284 | 4315 | 18161 | 53 | 22 |
|  | *A. stephensi* | 2703 | 8898 | 43449 | 78 | 298 |
|  | *B. cucurbitae* | 1734 | 33147 | 31273 | 33 | 94 |
|  | *B. dorsalis* | 1390 | 21519 | 23084 | 25 | 52 |
|  | *C. capitata* | 2547 | 72158 | 63923 | 46 | 173 |
|  | *C. quinquefasciatus* | 8334 | 43036 | 178633 | 832 | 246 |
|  | *D. ananassae* | 2454 | 11561 | 24611 | 45 | 21 |
|  | *D. erecta* | 2163 | 7651 | 16672 | 27 | 17 |
|  | *D. grimshawi* | 4616 | 27039 | 53644 | 87 | 42 |
|  | *D. melanogaster* | 1232 | 21296 | 11220 | 31 | 56 |
|  | *D. mojavensis* | 4474 | 34066 | 74505 | 71 | 39 |
|  | *D. persimilis* | 3591 | 24953 | 45336 | 106 | 64 |
|  | *D. pseudoobscura* | 3709 | 23208 | 47454 | 84 | 49 |
|  | *D. sechellia* | 1472 | 7477 | 15531 | 36 | 27 |
|  | *D. simulans* | 1280 | 6952 | 14452 | 33 | 17 |
|  | *D. virilis* | 4732 | 24044 | 53266 | 75 | 37 |
|  | *D. willistoni* | 4265 | 30546 | 64594 | 81 | 39 |
|  | *D. yakuba* | 2034 | 9413 | 19918 | 43 | 24 |
|  | *M. destructor* | 2667 | 12819 | 43611 | 350 | 267 |
|  | *M. scalaris* | 248 | 737 | 2383 | 22 | 6 |
|  | *M. domestica* | 1719 | 45673 | 50062 | 31 | 165 |
| Hemiptera | *A. pisum* | 1564 | 71555 | 54819 | 119 | 327 |
|  | *D. citri* | 311 | 56914 | 53248 | 74 | 149 |
|  | *N. lugens* | 808 | 8336 | 23054 | 60 | 31 |
|  | *R. prolixus* | 338 | 19968 | 71737 | 34 | 35 |
| Hymenoptera | *A. dorsata* | 687 | 58077 | 40226 | 65 | 184 |
|  | *A. florea* | 955 | 60729 | 43969 | 72 | 192 |
|  | *A. mellifera* | 2749 | 57316 | 91302 | 385 | 199 |
|  | *A. rosae* | 594 | 40922 | 30065 | 27 | 143 |
|  | *B. impatiens* | 556 | 18340 | 9296 | 22 | 120 |
|  | *B. terrestris* | 555 | 17166 | 8740 | 14 | 134 |
|  | *C. floridanus* | 2268 | 14398 | 59345 | 342 | 107 |
|  | *C. biroi* | 919 | 21729 | 10609 | 37 | 117 |
|  | *C. solmsi marchali* | 859 | 79197 | 86393 | 153 | 514 |
|  | *F. arisanus* | 498 | 6635 | 3639 | 18 | 32 |
|  | *H. saltator* | 7489 | 49201 | 237930 | 1246 | 346 |
|  | *L. humile* | 837 | 4695 | 27532 | 21 | 27 |
|  | *M. rotundata* | 628 | 9678 | 5574 | 24 | 42 |
|  | *M. demolitor* | 1360 | 45607 | 32848 | 97 | 338 |
|  | *N. vitripennis* | 1261 | 30398 | 54383 | 83 | 11 |
|  | *P. barbatus* | 880 | 9270 | 54756 | 52 | 47 |
|  | *V. emeryi* | 1060 | 23699 | 15166 | 23 | 167 |
|  | *W. auropunctata* | 1084 | 34719 | 23818 | 37 | 175 |
|  | *A. echinatior* | 2824 | 23842 | 76913 | 438 | 111 |
| Isoptera | *Z. nevadensis* | 627 | 13746 | 40283 | 43 | 20 |
| Lepidoptera | *B. mori* | 353 | 10250 | 62658 | 24 | 14 |
|  | *C. suppressalis* | 728 | 4577 | 7240 | 123 | 4 |
|  | *D. plexippus* | 374 | 8487 | 19258 | 29 | 14 |
|  | *H. melpomene* | 427 | 11099 | 19400 | 27 | 36 |
|  | *M. sexta* | 568 | 14445 | 20010 | 36 | 47 |
|  | *P. xylostella* | 7992 | 63034 | 120990 | 1406 | 227 |
