# Supplementary material for: Large-scale analysis reveals that the genome features of simple sequence repeats are generally conserved at the family level in insects
Source: BMC Genomics. 2017 Nov 6;18:848. doi: 10.1186/s12864-017-4234-0 (PMC5674736; doi:10.1186/s12864-017-4234-0)
Supplement: Supplementary file 9 — Table S8. Relative abundance of imperfect SSRs in different genomic regions. (DOCX 26 kb) [file 12864_2017_4234_MOESM9_ESM.docx]

**Table S8. The number of imperfect SSRs in different genome regions**

| **Order** | **Species** | **Exon** | **Intron** | **Intergenic regions** | **Spanning**  **exon-intron** | **Spanning intergenic-genetic** |
| --- | --- | --- | --- | --- | --- | --- |
| Anoplura | *P. humanus* | 962 | 15907 | 85276 | 70 | 44 |
| Coleoptera | *D. ponderosae* | 64 | 275 | 1081 | 8 | 1 |
|  | *T. castaneum* | 223 | 2085 | 2831 | 16 | 8 |
| Diptera | *A. aegypti* | 741 | 41592 | 13948 | 149 | 14 |
|  | *A. coluzzii* | 1343 | 6390 | 13640 | 44 | 28 |
|  | *A. darlingi* | 8392 | 12930 | 61858 | 1050 | 236 |
|  | *A. gambiae* | 1709 | 7660 | 20929 | 48 | 39 |
|  | *A. sinensis* | 935 | 1547 | 5145 | 22 | 8 |
|  | *A. stephensi* | 1422 | 2756 | 11520 | 34 | 190 |
|  | *B. cucurbitae* | 654 | 9783 | 8950 | 13 | 67 |
|  | *B. dorsalis* | 513 | 6472 | 6708 | 12 | 25 |
|  | *C. capitata* | 1315 | 41709 | 36595 | 27 | 115 |
|  | *C. quinquefasciatus* | 2005 | 10024 | 43273 | 451 | 150 |
|  | *D. ananassae* | 1369 | 3513 | 8289 | 28 | 5 |
|  | *D. erecta* | 1647 | 3009 | 7085 | 22 | 10 |
|  | *D. grimshawi* | 3828 | 20668 | 42305 | 85 | 28 |
|  | *D. melanogaster* | 969 | 8779 | 4351 | 25 | 39 |
|  | *D. mojavensis* | 3748 | 22532 | 49554 | 132 | 30 |
|  | *D. persimilis* | 2022 | 9336 | 16990 | 66 | 12 |
|  | *D. pseudoobscura* | 2518 | 9526 | 19880 | 53 | 16 |
|  | *D. sechellia* | 1055 | 2286 | 4797 | 27 | 9 |
|  | *D. simulans* | 973 | 2282 | 4936 | 24 | 7 |
|  | *D. virilis* | 3382 | 15469 | 35472 | 65 | 23 |
|  | *D. willistoni* | 2466 | 14766 | 32530 | 61 | 20 |
|  | *D. yakuba* | 1537 | 3688 | 8247 | 36 | 11 |
|  | *M. destructor* | 1216 | 4588 | 15499 | 169 | 148 |
|  | *M. scalaris* | 20 | 33 | 198 | 3 | 1 |
|  | *M. domestica* | 579 | 12472 | 14623 | 14 | 62 |
| Hemiptera | *A. pisum* | 785 | 22238 | 17170 | 45 | 184 |
|  | *D. citri* | 148 | 21648 | 24924 | 41 | 30 |
|  | *N. lugens* | 147 | 666 | 2049 | 13 | 4 |
|  | *R. prolixus* | 167 | 4070 | 16034 | 8 | 10 |
| Hymenoptera | *A. dorsata* | 396 | 25096 | 19254 | 54 | 150 |
|  | *A. florea* | 529 | 20645 | 16321 | 51 | 176 |
|  | *A. mellifera* | 1706 | 25009 | 41927 | 339 | 198 |
|  | *A. rosae* | 379 | 14046 | 11485 | 33 | 97 |
|  | *B. impatiens* | 334 | 9099 | 5164 | 21 | 140 |
|  | *B. terrestris* | 337 | 8012 | 4697 | 13 | 133 |
|  | *C. floridanus* | 1225 | 7626 | 32843 | 340 | 120 |
|  | *C. biroi* | 527 | 8337 | 4185 | 20 | 62 |
|  | *C. solmsi marchali* | 720 | 36038 | 40350 | 271 | 471 |
|  | *F. arisanus* | 246 | 2297 | 1298 | 14 | 22 |
|  | *H. saltator* | 4842 | 30759 | 150858 | 1531 | 472 |
|  | *L. humile* | 798 | 2029 | 14190 | 46 | 34 |
|  | *M. rotundata* | 390 | 3678 | 2063 | 11 | 42 |
|  | *M. demolitor* | 983 | 14700 | 11809 | 89 | 215 |
|  | *N. vitripennis* | 636 | 8832 | 16403 | 92 | 13 |
|  | *P. barbatus* | 980 | 3861 | 23535 | 67 | 50 |
|  | *V. emeryi* | 727 | 10272 | 6877 | 36 | 137 |
|  | *W. auropunctata* | 640 | 17173 | 12980 | 27 | 170 |
|  | *A. echinatior* | 1619 | 11584 | 38823 | 352 | 92 |
|  | *Z. nevadensis* | 252 | 2308 | 8518 | 18 | 7 |
| Isoptera | *B. mori* | 148 | 2500 | 14137 | 17 | 7 |
| Lepidoptera | *C. suppressalis* | 133 | 769 | 1344 | 34 | 2 |
|  | *D. plexippus* | 179 | 2697 | 5468 | 11 | 4 |
|  | *H. melpomene* | 144 | 2216 | 4206 | 18 | 9 |
|  | *M. sexta* | 296 | 2879 | 4128 | 4 | 20 |
|  | *P. xylostella* | 1708 | 11867 | 23129 | 456 | 64 |
